# Supplementary material for: On the role of financial support programs in mitigating the SARS-CoV-2 spread in Brazil
Source: BMC Public Health. 2022 Sep 20;22:1781. doi: 10.1186/s12889-022-14155-z (PMC9485798; doi:10.1186/s12889-022-14155-z)
Supplement: Supplementary file 1 — The Supplementary Materials contain additional figures and tables. [file 12889_2022_14155_MOESM1_ESM.pdf]

Supplementary Material to: On the Role of Financial Support Programs in Mitigating the Sars-CoV-2 Spread in Brazil

Vinicius V. L. Albani[1], Roseane Albani[2], Nara Bobko[3], Eduardo Massad[4 5] and Jorge P. Zubelli[6]

June 21, 2022

[1]Department of Mathematics, Federal University of Santa Catarina, Florianopolis, Brazil, v.albani@ufsc.br

[2]Instituto Politecnico do Rio de Janeiro, State University of Rio de Janeiro, Nova Friburgo, Brazil, roseanealves75@gmail.com

[3]Federal University of Technology - Paraná, Curitiba, Brazil, narabobko@gmail.com

[4]School of Medicine, University of São Paulo and LIM01-HCFMUSP, São Paulo, Brazil

[5]School of Applied Mathematics, Fundação Getúlio Vargas, Rio de Janeiro, Brazil, eduardo.massad@fgv.br

[6]Mathematics Department, Khalifa University, Abu Dhabi, UAE, jorge.zubelli@ku.ac.ae

1 North Region

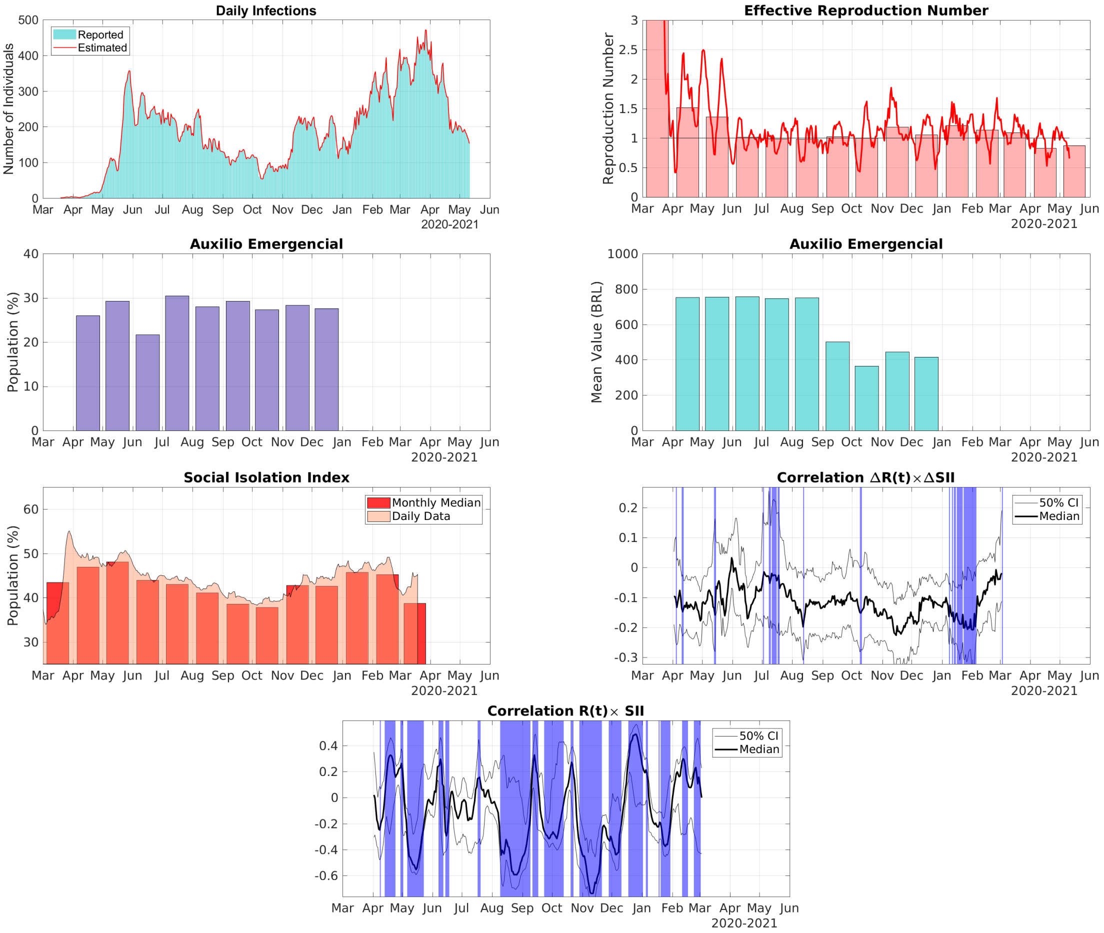

Figure S.1: Data for the State of Acre in the North Region of Brazil. Top Row, Right: comparison between the 7-day moving average of daily reports of infections and model predictions (right). Top Row, Left: The solid line represents the time-dependent reproduction number and the bars are the corresponding monthly median values. Middle Row, Right: The bars represent the proportions of the State population receiving the Auxílio Emergencial by month. Middle Row, Left: The bars represent the statewide average amount paid by Auxílio Emergencial each month. Bottom Row, Left: The area graph is the 7-day moving average of the social isolation index and the bars represent the corresponding monthly median values. Bottom Row, Right: Correlation between the daily increments of the social isolation index and the reproduction number. The marked regions in the correlation plot indicate that the estimated values are significantly different from zero, for a 10% p-value.

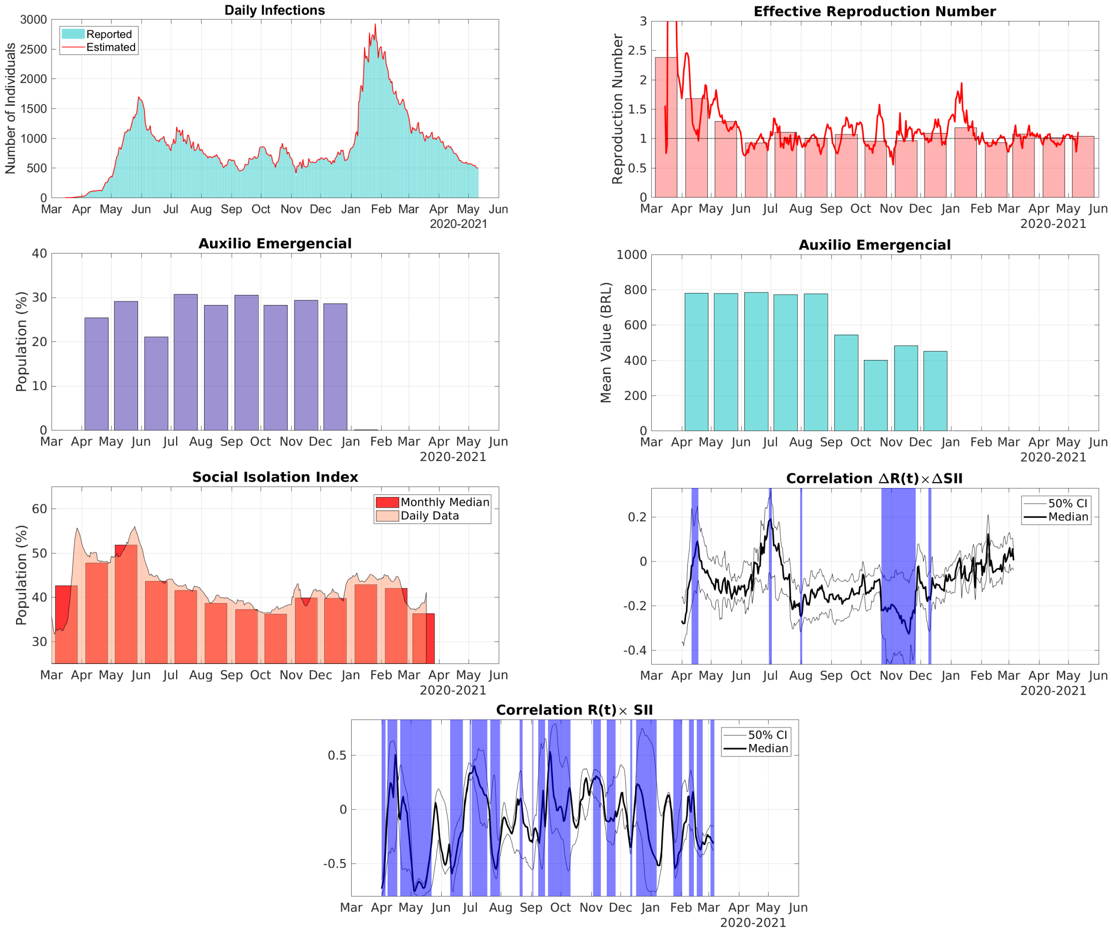

Figure S.2: Data for the State of Amazonas in the North Region of Brazil. Top Row, Right: comparison between the 7-day moving average of daily reports of infections and model predictions (right). Top Row, Left: The solid line represents the time-dependent reproduction number and the bars are the corresponding monthly median values. Middle Row, Right: The bars represent the proportions of the State population receiving the Auxílio Emergencial by month. Middle Row, Left: The bars represent the statewide average amount paid by Auxílio Emergencial each month. Bottom Row, Left: The area graph is the 7-day moving average of the social isolation index and the bars represent the corresponding monthly median values. Bottom Row, Right: Correlation between the daily increments of the social isolation index and the reproduction number. The marked regions in the correlation plot indicate that the estimated values are significantly different from zero, for a 10% p-value.

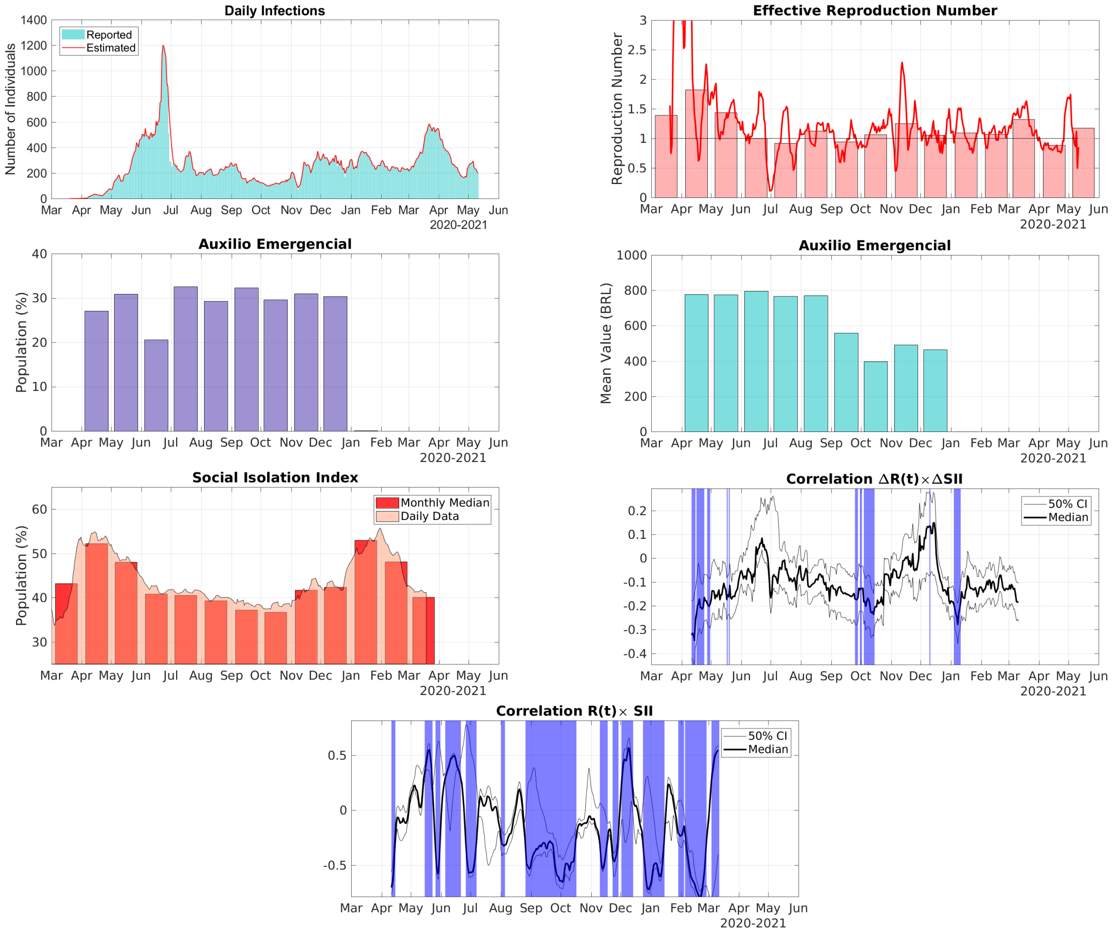

Figure S.3: Data for the State of Amapá in the North Region of Brazil. Top Row, Right: comparison between the 7-day moving average of daily reports of infections and model predictions (right). Top Row, Left: The solid line represents the time-dependent reproduction number and the bars are the corresponding monthly median values. Middle Row, Right: The bars represent the proportions of the State population receiving the Auxílio Emergencial by month. Middle Row, Left: The bars represent the statewide average amount paid by Auxílio Emergencial each month. Bottom Row, Left: The area graph is the 7-day moving average of the social isolation index and the bars represent the corresponding monthly median values. Bottom Row, Right: Correlation between the daily increments of the social isolation index and the reproduction number. The marked regions in the correlation plot indicate that the estimated values are significantly different from zero, for a 10% p-value.

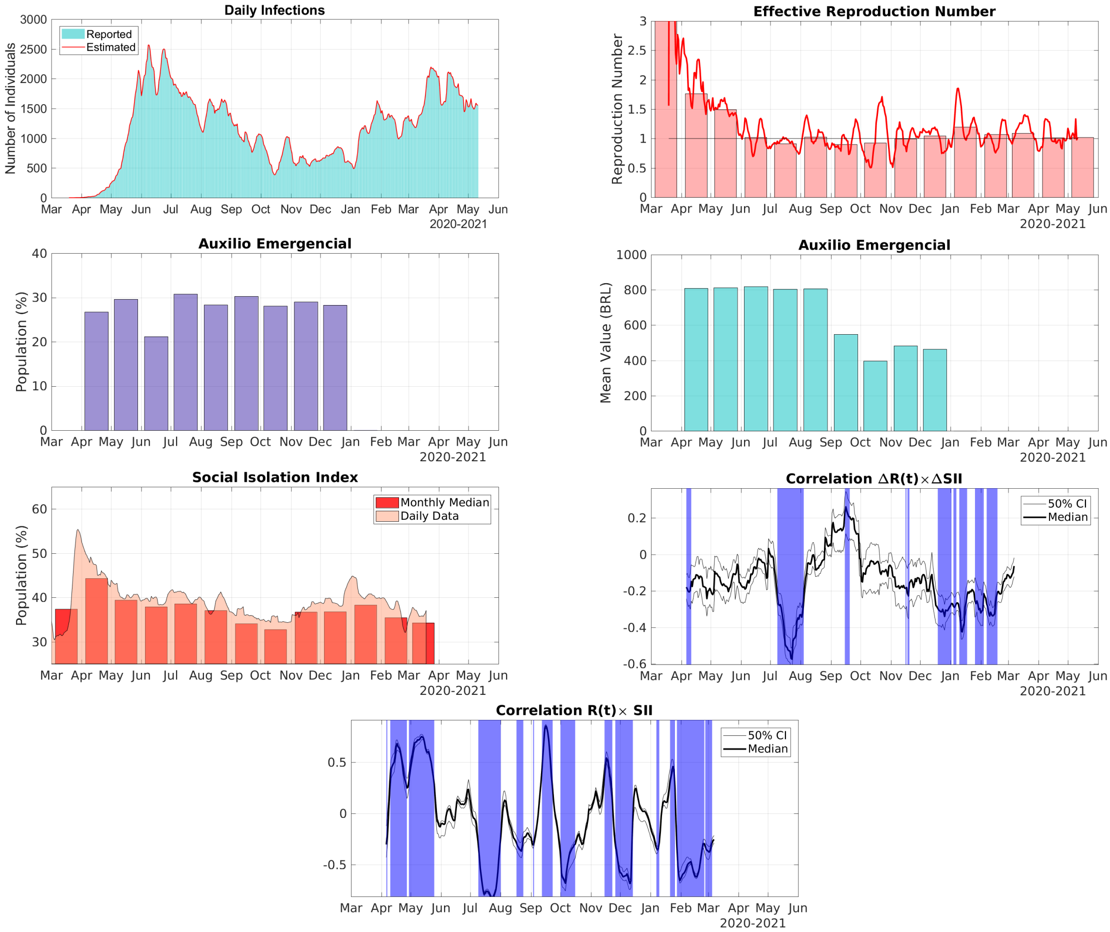

Figure S.4: Data for the State of Pará in the North Region of Brazil. Top Row, Right: comparison between the 7-day moving average of daily reports of infections and model predictions (right). Top Row, Left: The solid line represents the time-dependent reproduction number and the bars are the corresponding monthly median values. Middle Row, Right: The bars represent the proportions of the State population receiving the Auxílio Emergencial by month. Middle Row, Left: The bars represent the statewide average amount paid by Auxílio Emergencial each month. Bottom Row, Left: The area graph is the 7-day moving average of the social isolation index and the bars represent the corresponding monthly median values. Bottom Row, Right: Correlation between the daily increments of the social isolation index and the reproduction number. The marked regions in the correlation plot indicate that the estimated values are significantly different from zero, for a 10% p-value.

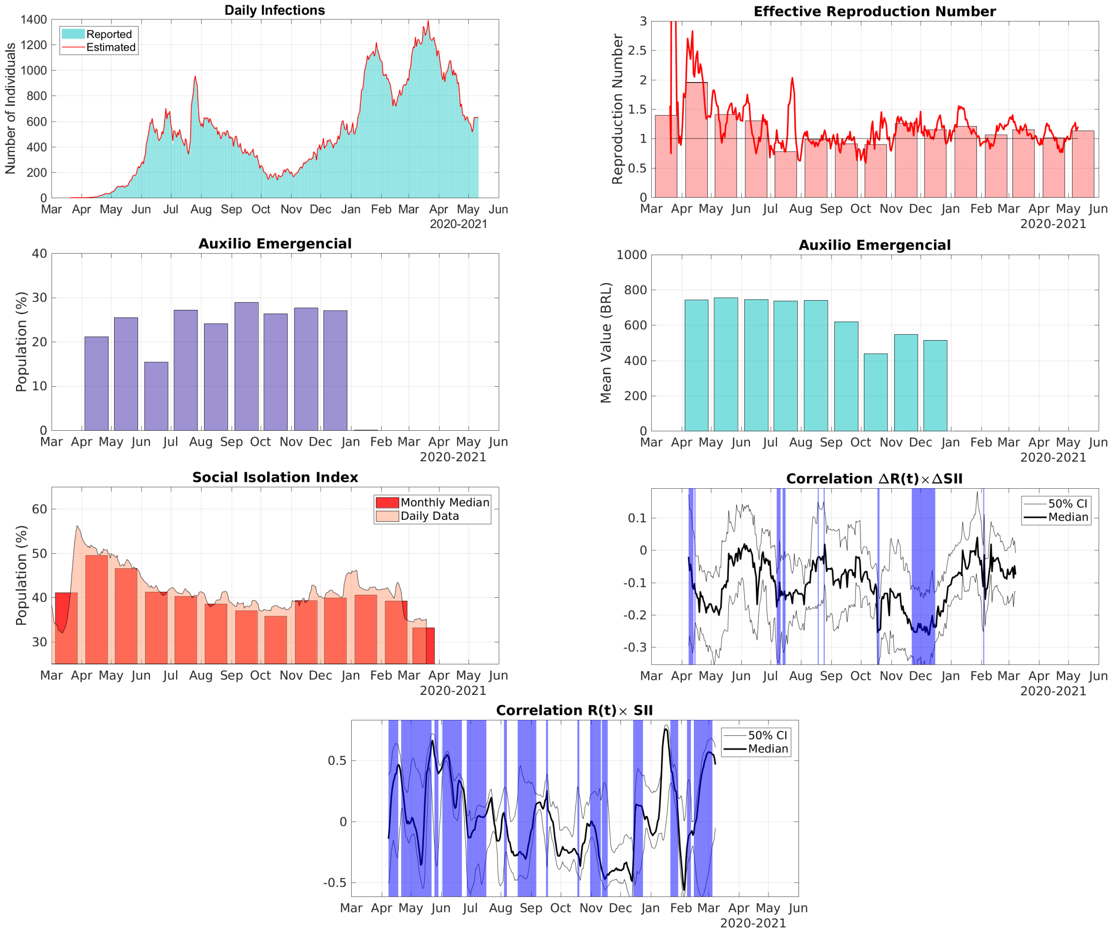

Figure S.5: Data for the State of Rondônia in the North Region of Brazil. Top Row, Right: comparison between the 7-day moving average of daily reports of infections and model predictions (right). Top Row, Left: The solid line represents the time-dependent reproduction number and the bars are the corresponding monthly median values. Middle Row, Right: The bars represent the proportions of the State population receiving the Auxílio Emergencial by month. Middle Row, Left: The bars represent the statewide average amount paid by Auxílio Emergencial each month. Bottom Row, Left: The area graph is the 7-day moving average of the social isolation index and the bars represent the corresponding monthly median values. Bottom Row, Right: Correlation between the daily increments of the social isolation index and the reproduction number. The marked regions in the correlation plot indicate that the estimated values are significantly different from zero, for a 10% p-value.

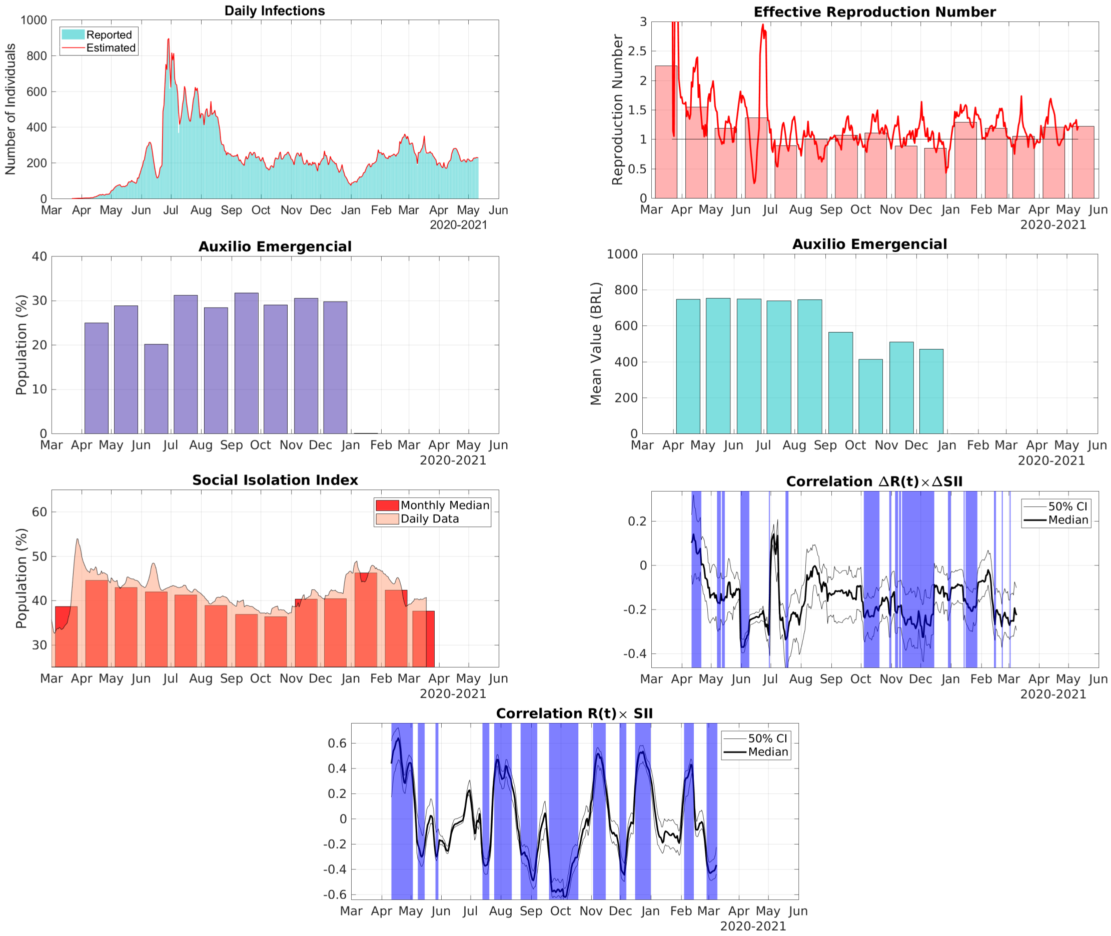

Figure S.6: Data for the State of Roraima in the North Region of Brazil. Top Row, Right: comparison between the 7-day moving average of daily reports of infections and model predictions (right). Top Row, Left: The solid line represents the time-dependent reproduction number and the bars are the corresponding monthly median values. Middle Row, Right: The bars represent the proportions of the State population receiving the Auxílio Emergencial by month. Middle Row, Left: The bars represent the statewide average amount paid by Auxílio Emergencial each month. Bottom Row, Left: The area graph is the 7-day moving average of the social isolation index and the bars represent the corresponding monthly median values. Bottom Row, Right: Correlation between the daily increments of the social isolation index and the reproduction number. The marked regions in the correlation plot indicate that the estimated values are significantly different from zero, for a 10% p-value.

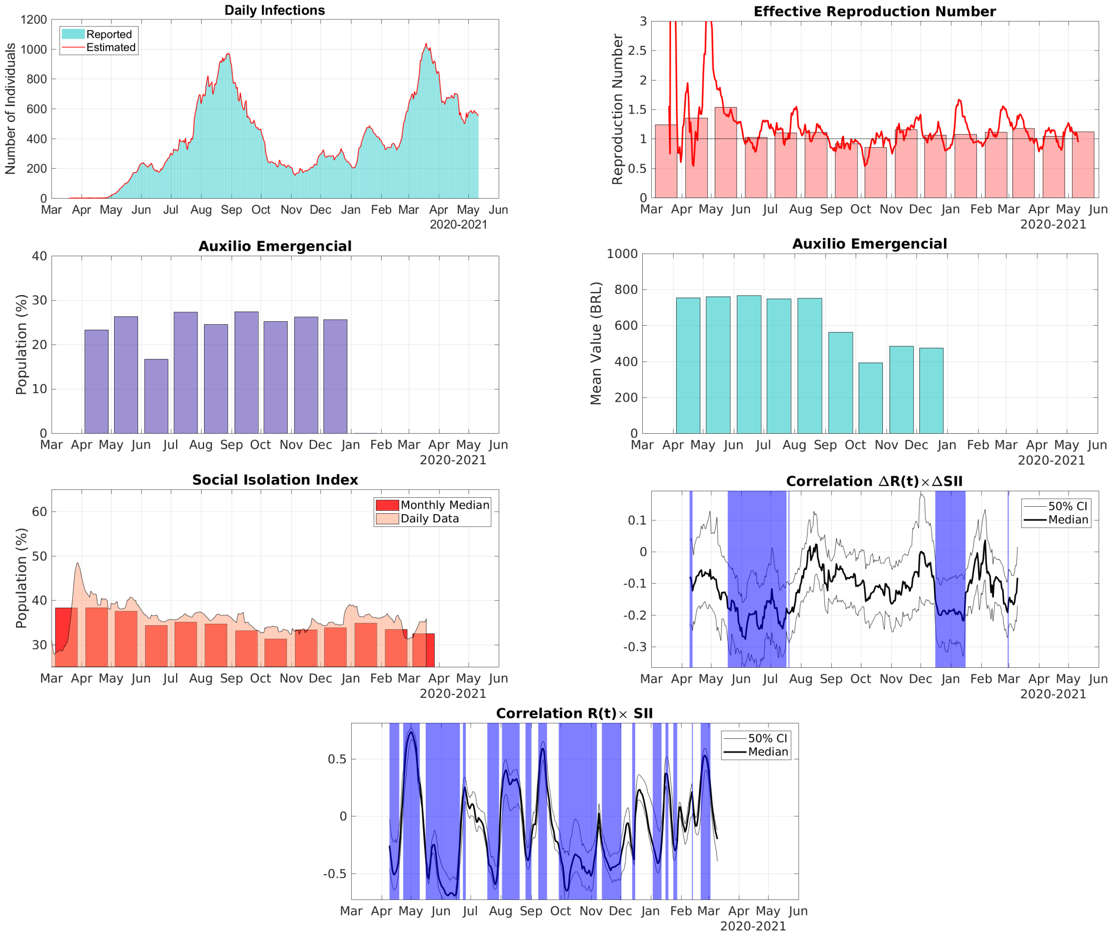

Figure S.7: Data for the State of Tocantins in the North Region of Brazil. Top Row, Right: comparison between the 7-day moving average of daily reports of infections and model predictions (right). Top Row, Left: The solid line represents the time-dependent reproduction number and the bars are the corresponding monthly median values. Middle Row, Right: The bars represent the proportions of the State population receiving the Auxílio Emergencial by month. Middle Row, Left: The bars represent the statewide average amount paid by Auxílio Emergencial each month. Bottom Row, Left: The area graph is the 7-day moving average of the social isolation index and the bars represent the corresponding monthly median values. Bottom Row, Right: Correlation between the daily increments of the social isolation index and the reproduction number. The marked regions in the correlation plot indicate that the estimated values are significantly different from zero, for a 10% p-value.

2 Northeast Region

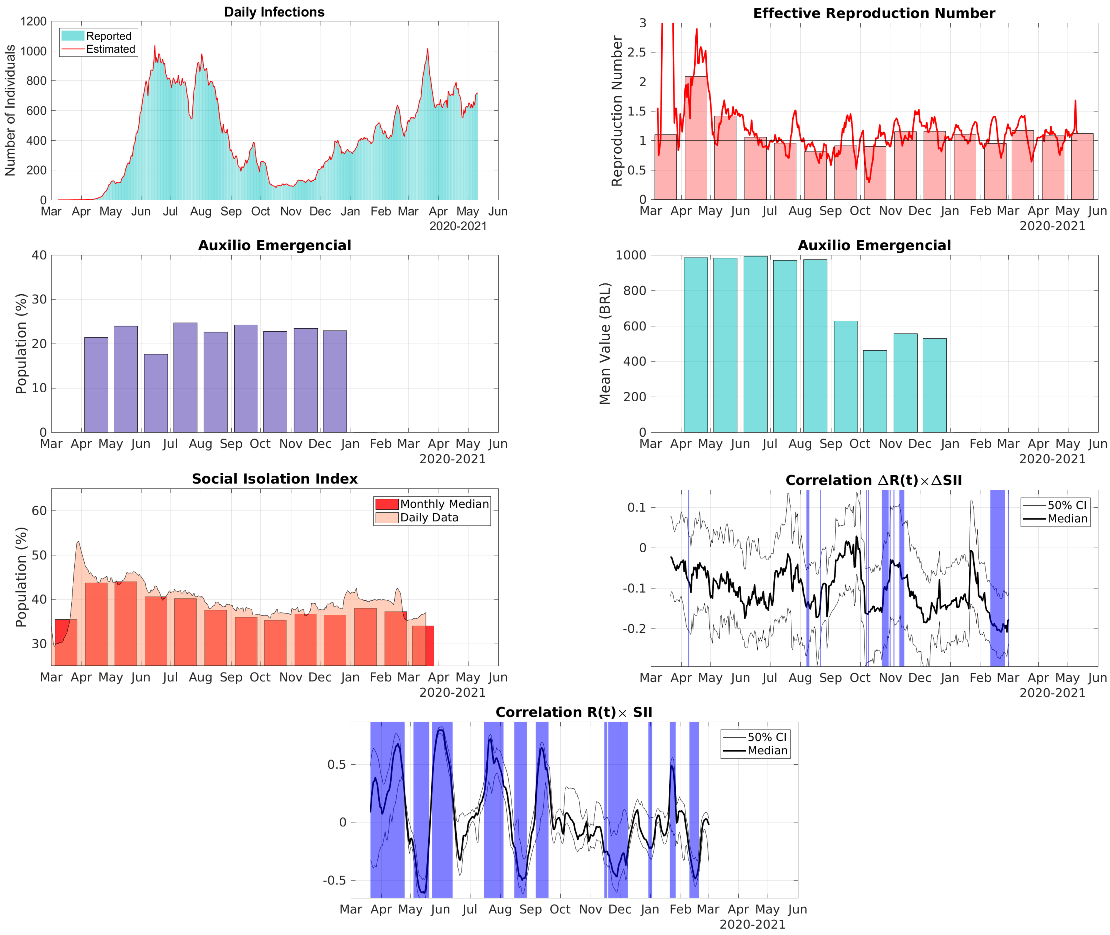

Figure S.8: Data for the State of Alagoas in the Northeast Region of Brazil. Top Row, Right: comparison between the 7-day moving average of daily reports of infections and model predictions (right). Top Row, Left: The solid line represents the time-dependent reproduction number and the bars are the corresponding monthly median values. Middle Row, Right: The bars represent the proportions of the State population receiving the Auxílio Emergencial by month. Middle Row, Left: The bars represent the statewide average amount paid by Auxílio Emergencial each month. Bottom Row, Left: The area graph is the 7-day moving average of the social isolation index and the bars represent the corresponding monthly median values. Bottom Row, Right: Correlation between the daily increments of the social isolation index and the reproduction number. The marked regions in the correlation plot indicate that the estimated values are significantly different from zero, for a 10% p-value.

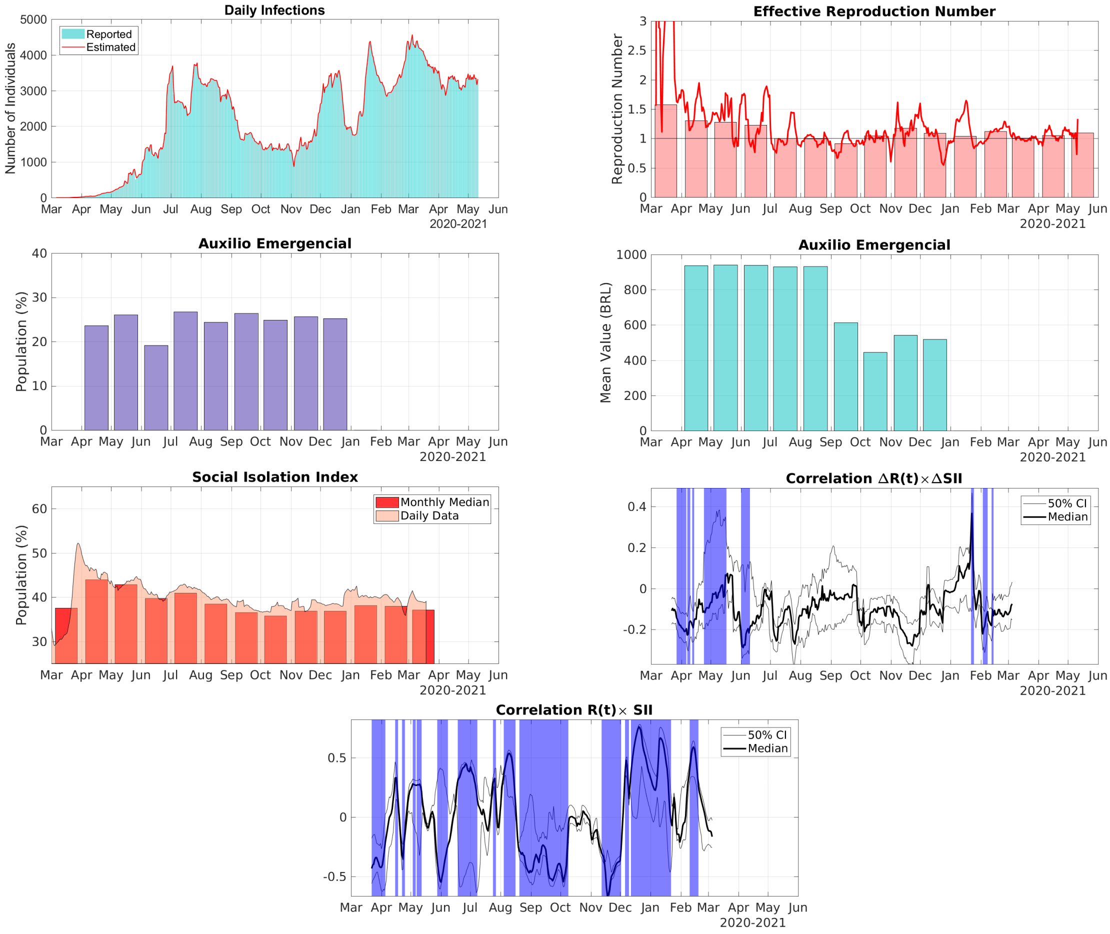

Figure S.9: Data for the State of Bahia in the Northeast Region of Brazil. Top Row, Right: comparison between the 7-day moving average of daily reports of infections and model predictions (right). Top Row, Left: The solid line represents the time-dependent reproduction number and the bars are the corresponding monthly median values. Middle Row, Right: The bars represent the proportions of the State population receiving the Auxílio Emergencial by month. Middle Row, Left: The bars represent the statewide average amount paid by Auxílio Emergencial each month. Bottom Row, Left: The area graph is the 7-day moving average of the social isolation index and the bars represent the corresponding monthly median values. Bottom Row, Right: Correlation between the daily increments of the social isolation index and the reproduction number. The marked regions in the correlation plot indicate that the estimated values are significantly different from zero, for a 10% p-value.

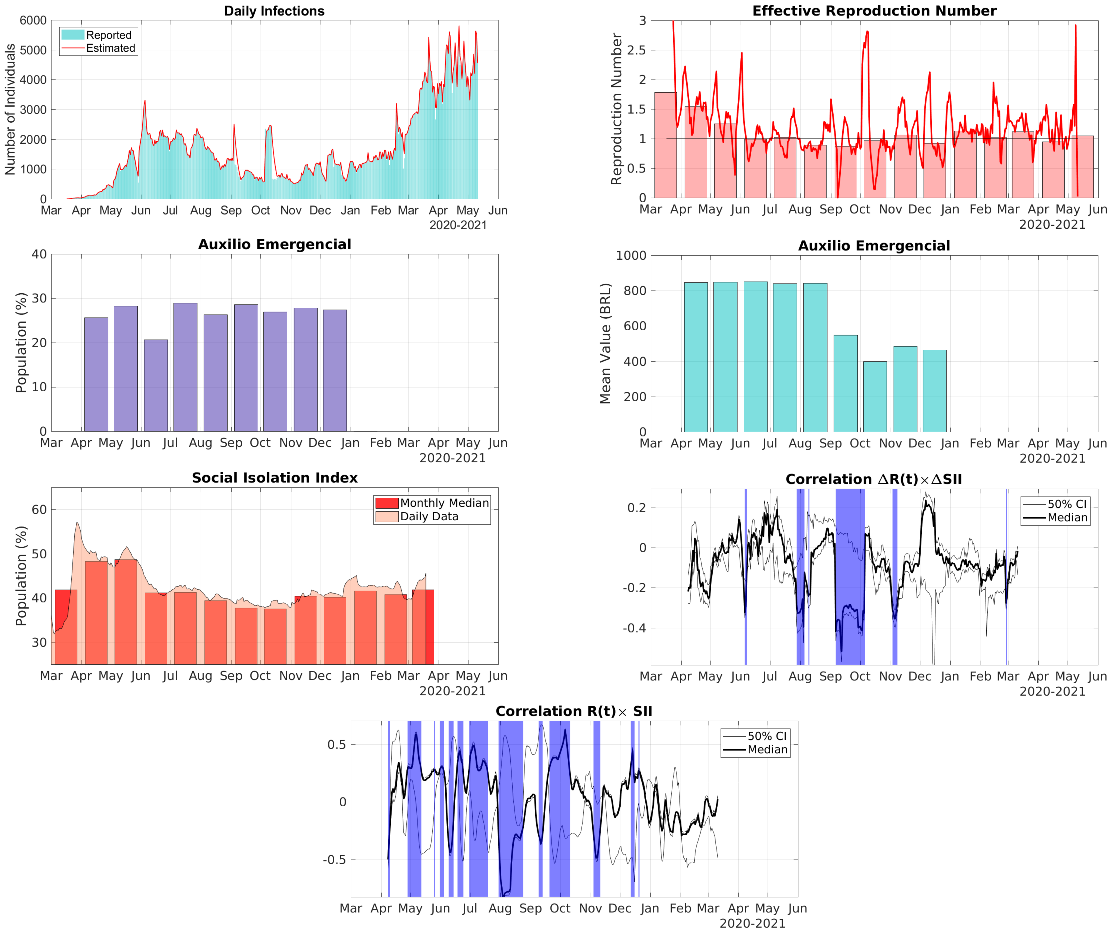

Figure S.10: Data for the State of Ceará in the Northeast Region of Brazil. Top Row, Right: comparison between the 7-day moving average of daily reports of infections and model predictions (right). Top Row, Left: The solid line represents the time-dependent reproduction number and the bars are the corresponding monthly median values. Middle Row, Right: The bars represent the proportions of the State population receiving the Auxílio Emergencial by month. Middle Row, Left: The bars represent the statewide average amount paid by Auxílio Emergencial each month. Bottom Row, Left: The area graph is the 7-day moving average of the social isolation index and the bars represent the corresponding monthly median values. Bottom Row, Right: Correlation between the daily increments of the social isolation index and the reproduction number. The marked regions in the correlation plot indicate that the estimated values are significantly different from zero, for a 10% p-value.

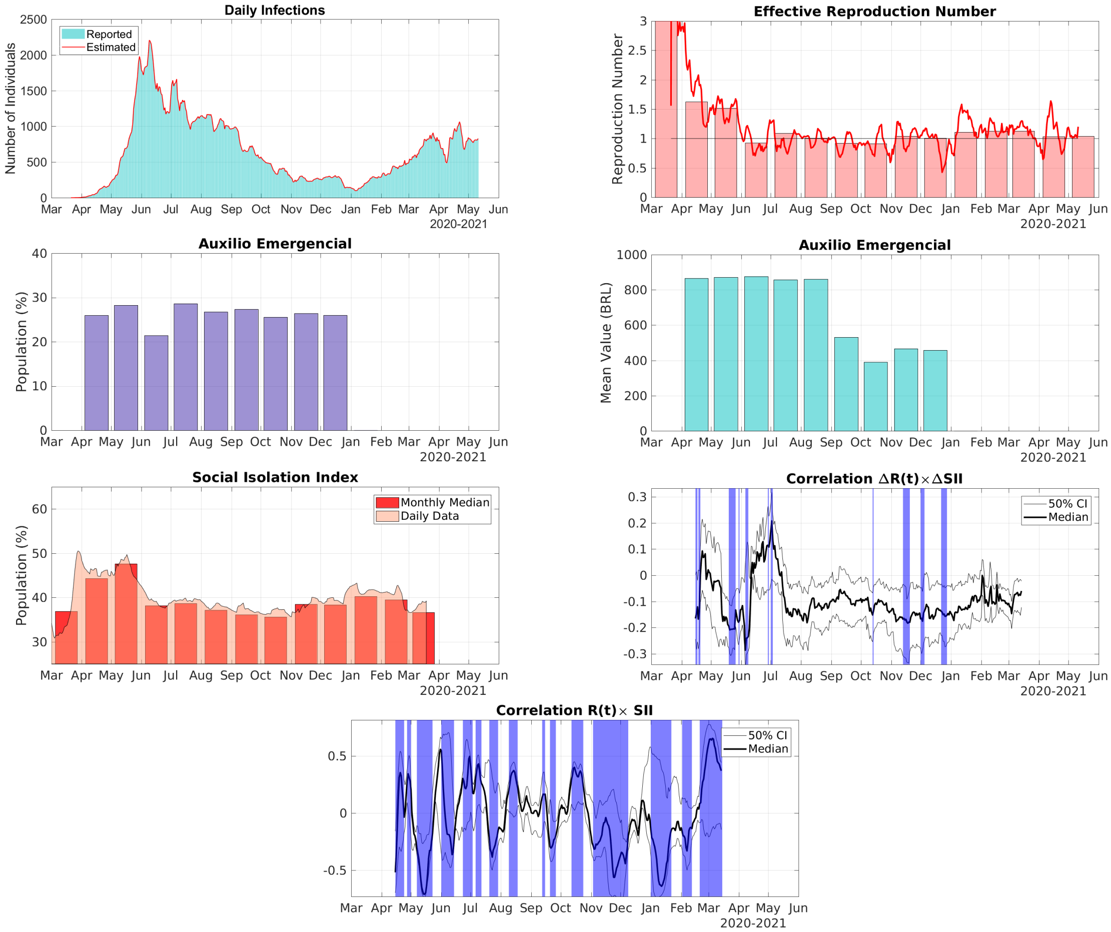

Figure S.11: Data for the State of Maranhão in the Northeast Region of Brazil. Top Row, Right: comparison between the 7-day moving average of daily reports of infections and model predictions (right). Top Row, Left: The solid line represents the time-dependent reproduction number and the bars are the corresponding monthly median values. Middle Row, Right: The bars represent the proportions of the State population receiving the Auxílio Emergencial by month. Middle Row, Left: The bars represent the statewide average amount paid by Auxílio Emergencial each month. Bottom Row, Left: The area graph is the 7-day moving average of the social isolation index and the bars represent the corresponding monthly median values. Bottom Row, Right: Correlation between the daily increments of the social isolation index and the reproduction number. The marked regions in the correlation plot indicate that the estimated values are significantly different from zero, for a 10% p-value.

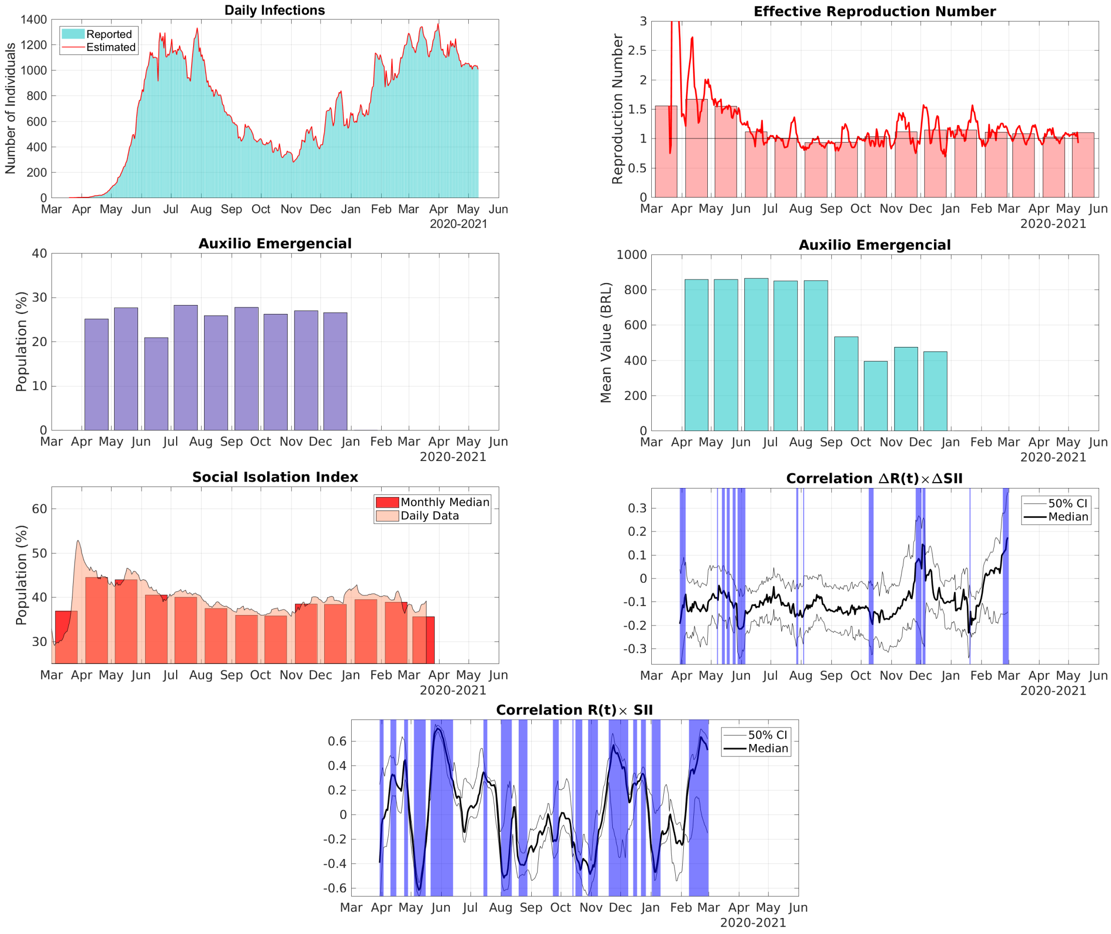

Figure S.12: Data for the State of Paraíba in the Northeast Region of Brazil. Top Row, Right: comparison between the 7-day moving average of daily reports of infections and model predictions (right). Top Row, Left: The solid line represents the time-dependent reproduction number and the bars are the corresponding monthly median values. Middle Row, Right: The bars represent the proportions of the State population receiving the Auxílio Emergencial by month. Middle Row, Left: The bars represent the statewide average amount paid by Auxílio Emergencial each month. Bottom Row, Left: The area graph is the 7-day moving average of the social isolation index and the bars represent the corresponding monthly median values. Bottom Row, Right: Correlation between the daily increments of the social isolation index and the reproduction number. The marked regions in the correlation plot indicate that the estimated values are significantly different from zero, for a 10% p-value.

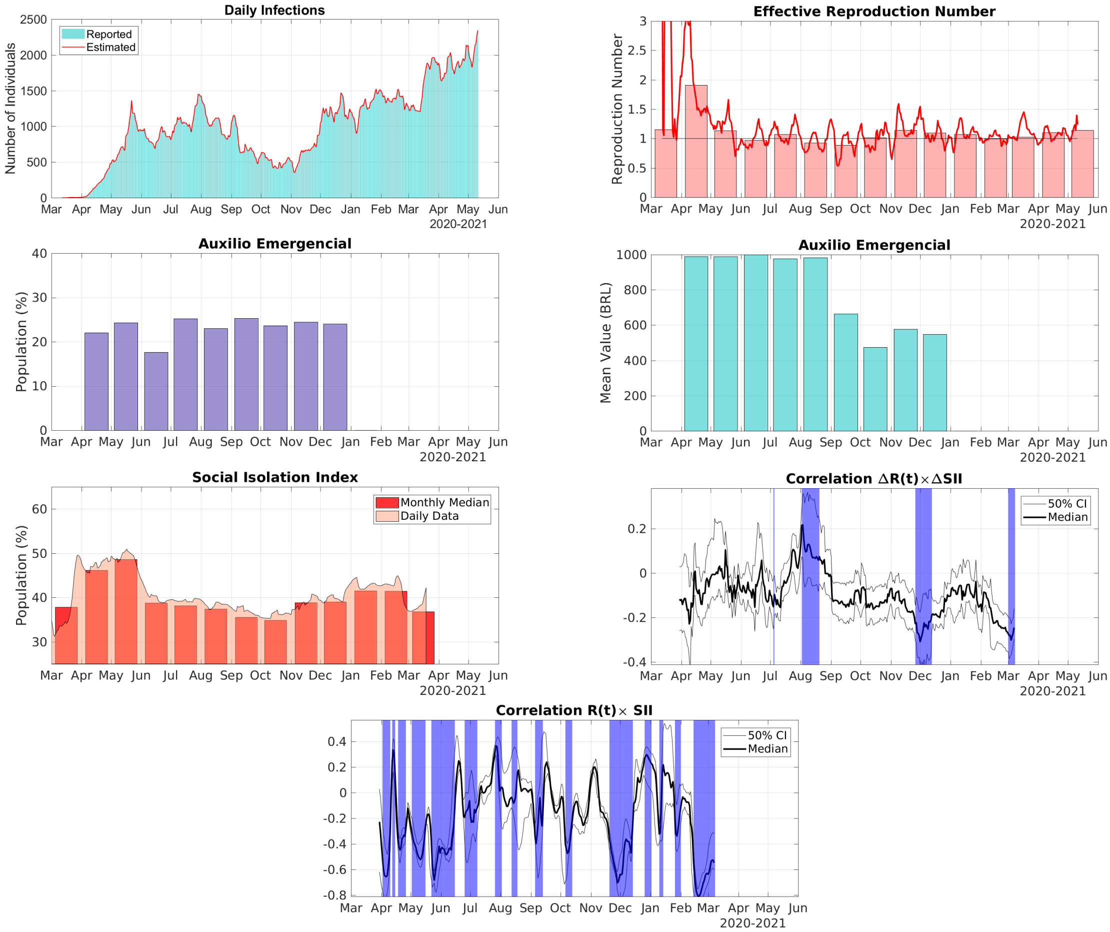

Figure S.13: Data for the State of Pernambuco in the Northeast Region of Brazil. Top Row, Right: comparison between the 7-day moving average of daily reports of infections and model predictions (right). Top Row, Left: The solid line represents the time-dependent reproduction number and the bars are the corresponding monthly median values. Middle Row, Right: The bars represent the proportions of the State population receiving the Auxílio Emergencial by month. Middle Row, Left: The bars represent the statewide average amount paid by Auxílio Emergencial each month. Bottom Row, Left: The area graph is the 7-day moving average of the social isolation index and the bars represent the corresponding monthly median values. Bottom Row, Right: Correlation between the daily increments of the social isolation index and the reproduction number. The marked regions in the correlation plot indicate that the estimated values are significantly different from zero, for a 10% p-value.

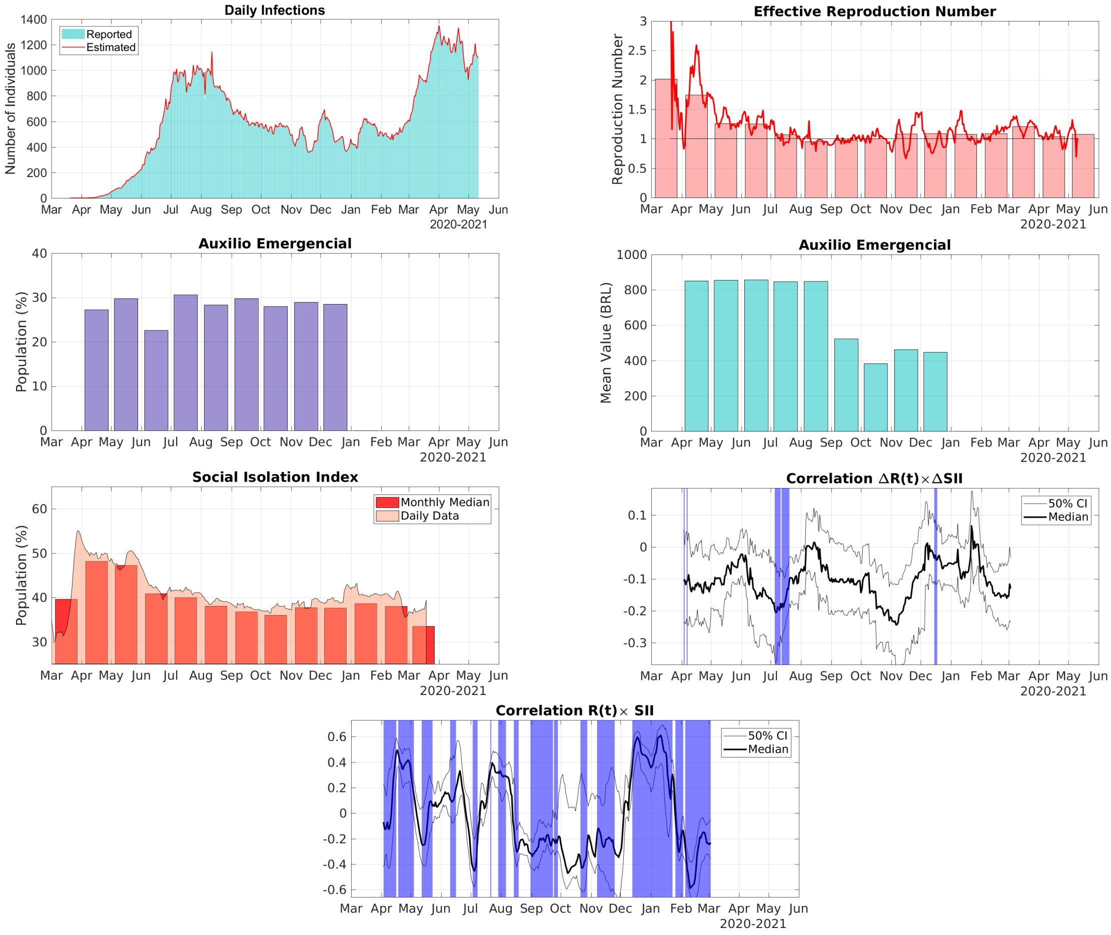

Figure S.14: Data for the State of Piauí in the Northeast Region of Brazil. Top Row, Right: comparison between the 7-day moving average of daily reports of infections and model predictions (right). Top Row, Left: The solid line represents the time-dependent reproduction number and the bars are the corresponding monthly median values. Middle Row, Right: The bars represent the proportions of the State population receiving the Auxílio Emergencial by month. Middle Row, Left: The bars represent the statewide average amount paid by Auxílio Emergencial each month. Bottom Row, Left: The area graph is the 7-day moving average of the social isolation index and the bars represent the corresponding monthly median values. Bottom Row, Right: Correlation between the daily increments of the social isolation index and the reproduction number. The marked regions in the correlation plot indicate that the estimated values are significantly different from zero, for a 10% p-value.

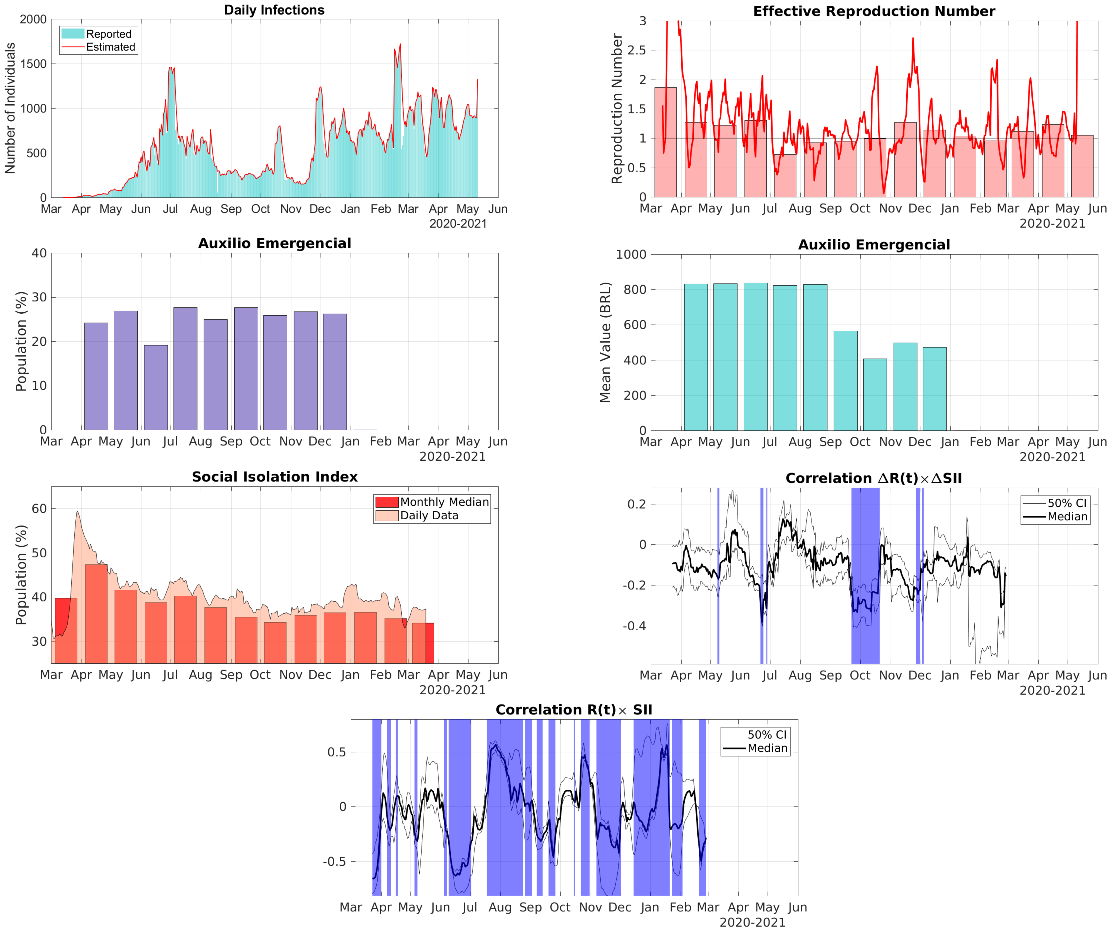

Figure S.15: Data for the State of Rio Grande do Norte in the Northeast Region of Brazil. Top Row, Right: comparison between the 7-day moving average of daily reports of infections and model predictions (right). Top Row, Left: The solid line represents the time-dependent reproduction number and the bars are the corresponding monthly median values. Middle Row, Right: The bars represent the proportions of the State population receiving the Auxílio Emergencial by month. Middle Row, Left: The bars represent the statewide average amount paid by Auxílio Emergencial each month. Bottom Row, Left: The area graph is the 7-day moving average of the social isolation index and the bars represent the corresponding monthly median values. Bottom Row, Right: Correlation between the daily increments of the social isolation index and the reproduction number. The marked regions in the correlation plot indicate that the estimated values are significantly different from zero, for a 10% p-value.

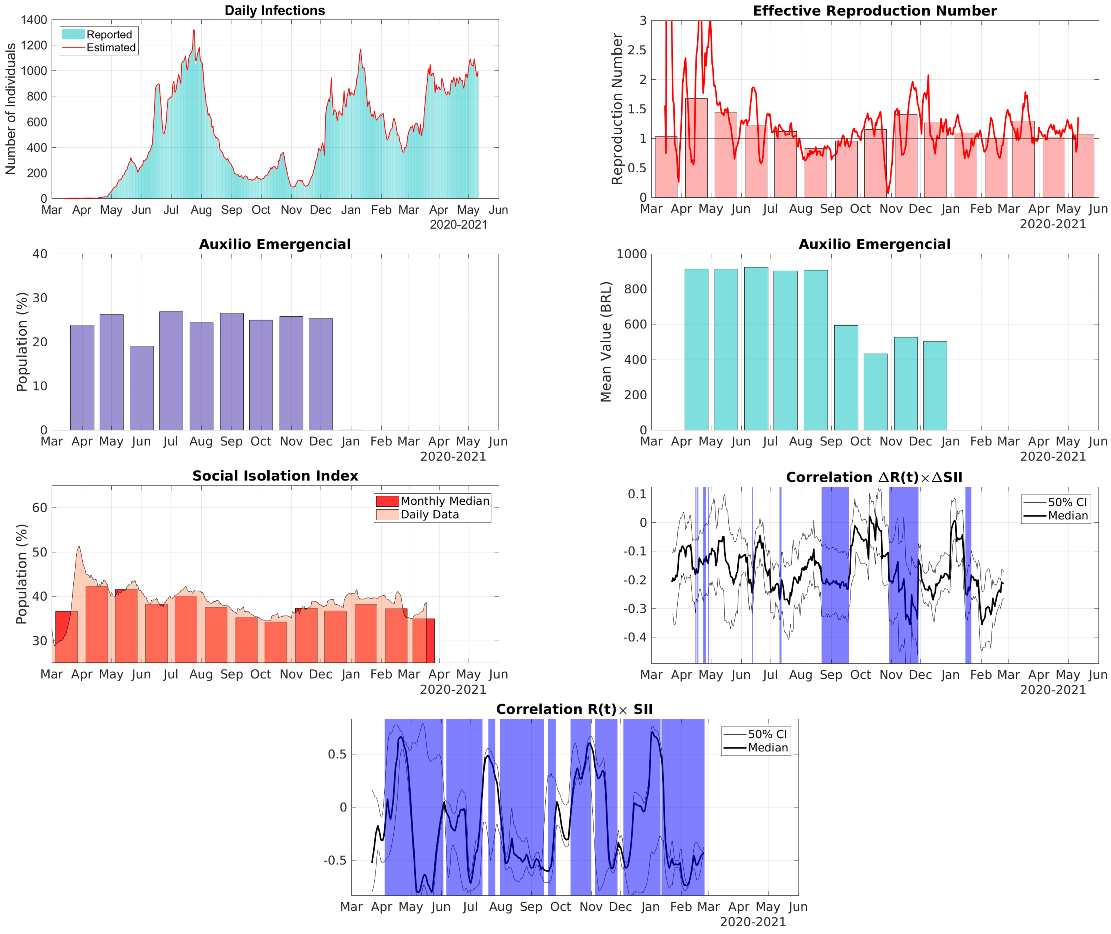

Figure S.16: Data for the State of Sergipe in the Northeast Region of Brazil. Top Row, Right: comparison between the 7-day moving average of daily reports of infections and model predictions (right). Top Row, Left: The solid line represents the time-dependent reproduction number and the bars are the corresponding monthly median values. Middle Row, Right: The bars represent the proportions of the State population receiving the Auxílio Emergencial by month. Middle Row, Left: The bars represent the statewide average amount paid by Auxílio Emergencial each month. Bottom Row, Left: The area graph is the 7-day moving average of the social isolation index and the bars represent the corresponding monthly median values. Bottom Row, Right: Correlation between the daily increments of the social isolation index and the reproduction number. The marked regions in the correlation plot indicate that the estimated values are significantly different from zero, for a 10% p-value.

3 Central-West Region

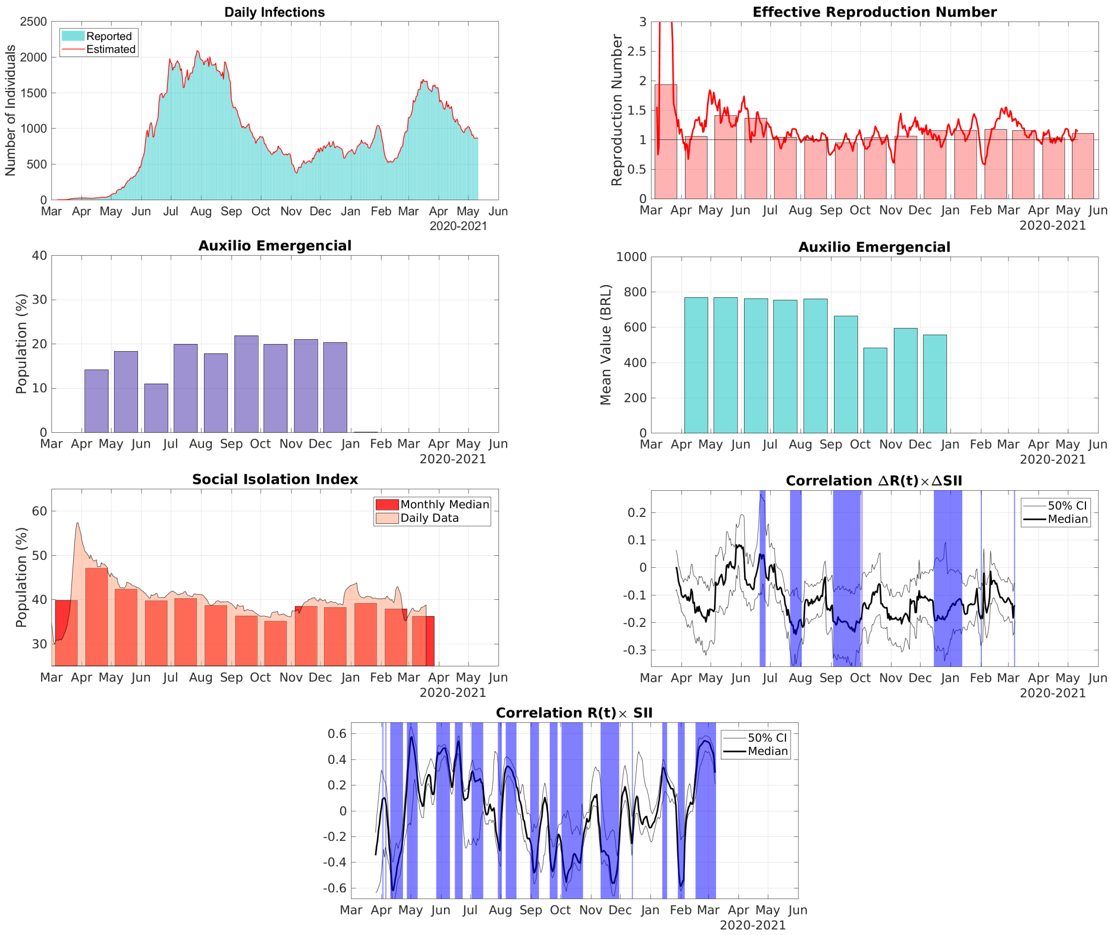

Figure S.17: Data for the Distrito Federal in the Central-West Region of Brazil. Top Row, Right: comparison between the 7-day moving average of daily reports of infections and model predictions (right). Top Row, Left: The solid line represents the time-dependent reproduction number and the bars are the corresponding monthly median values. Middle Row, Right: The bars represent the proportions of the State population receiving the Auxílio Emergencial by month. Middle Row, Left: The bars represent the statewide average amount paid by Auxílio Emergencial each month. Bottom Row, Left: The area graph is the 7-day moving average of the social isolation index and the bars represent the corresponding monthly median values. Bottom Row, Right: Correlation between the daily increments of the social isolation index and the reproduction number. The marked regions in the correlation plot indicate that the estimated values are significantly different from zero, for a 10% p-value.

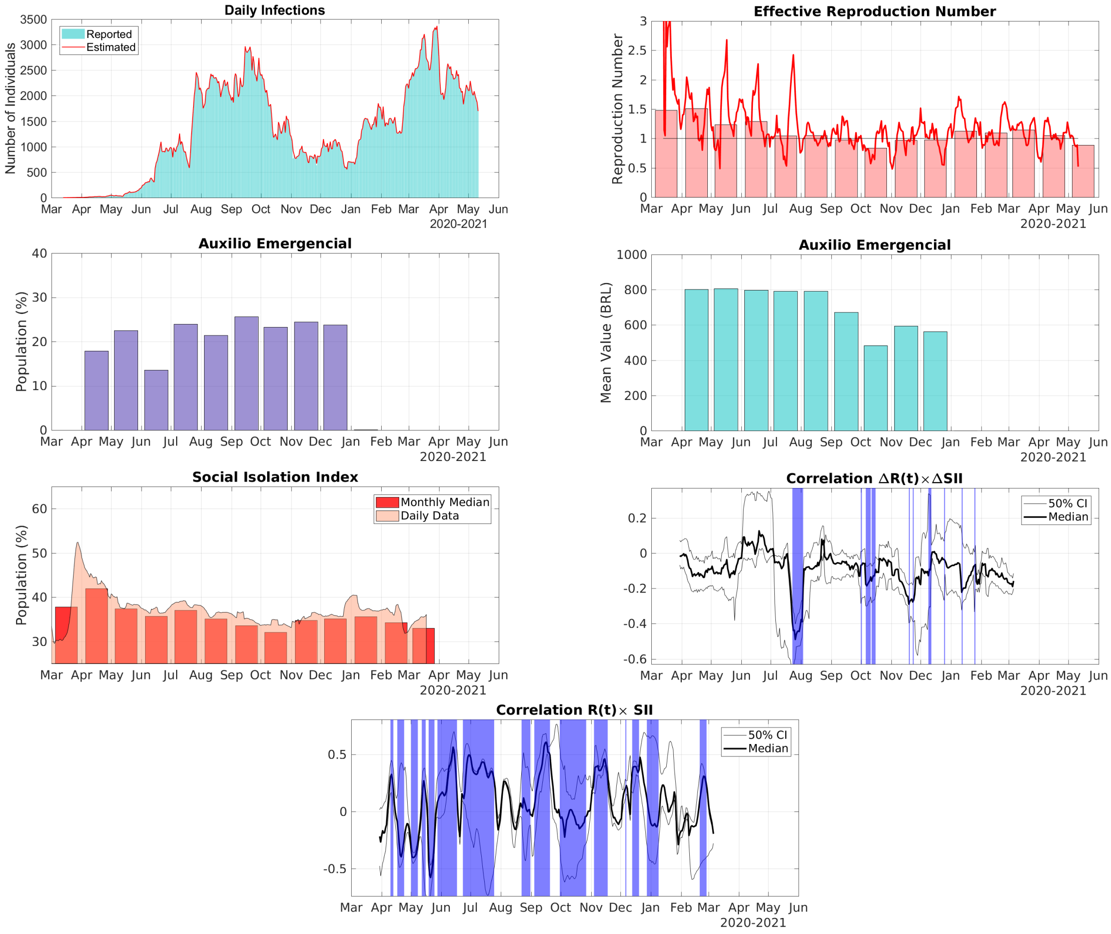

Figure S.18: Data for the State of Goiás in the Central-West Region of Brazil. Top Row, Right: comparison between the 7-day moving average of daily reports of infections and model predictions (right). Top Row, Left: The solid line represents the time-dependent reproduction number and the bars are the corresponding monthly median values. Middle Row, Right: The bars represent the proportions of the State population receiving the Auxílio Emergencial by month. Middle Row, Left: The bars represent the statewide average amount paid by Auxílio Emergencial each month. Bottom Row, Left: The area graph is the 7-day moving average of the social isolation index and the bars represent the corresponding monthly median values. Bottom Row, Right: Correlation between the daily increments of the social isolation index and the reproduction number. The marked regions in the correlation plot indicate that the estimated values are significantly different from zero, for a 10% p-value.

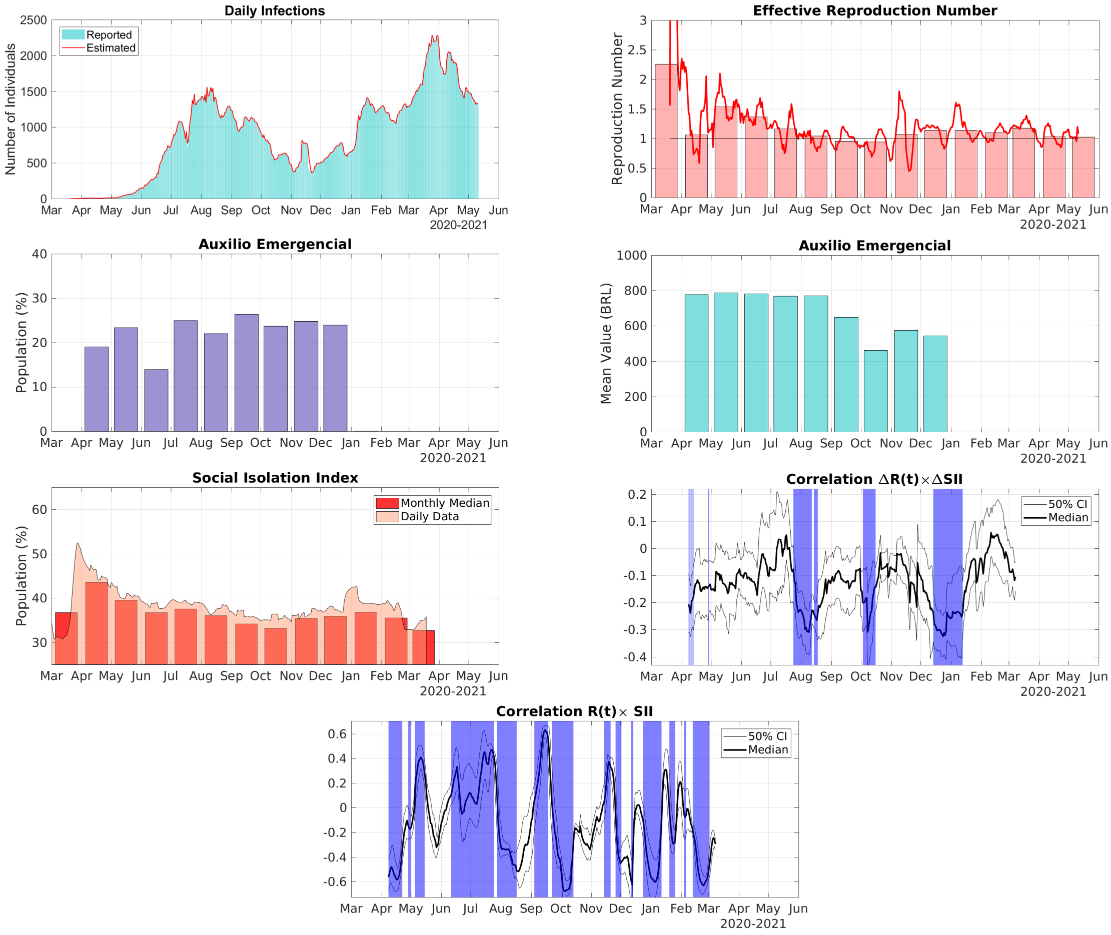

Figure S.19: Data for the State of Mato Grosso in the Central-West Region of Brazil. Top Row, Right: comparison between the 7-day moving average of daily reports of infections and model predictions (right). Top Row, Left: The solid line represents the time-dependent reproduction number and the bars are the corresponding monthly median values. Middle Row, Right: The bars represent the proportions of the State population receiving the Auxílio Emergencial by month. Middle Row, Left: The bars represent the statewide average amount paid by Auxílio Emergencial each month. Bottom Row, Left: The area graph is the 7-day moving average of the social isolation index and the bars represent the corresponding monthly median values. Bottom Row, Right: Correlation between the daily increments of the social isolation index and the reproduction number. The marked regions in the correlation plot indicate that the estimated values are significantly different from zero, for a 10% p-value.

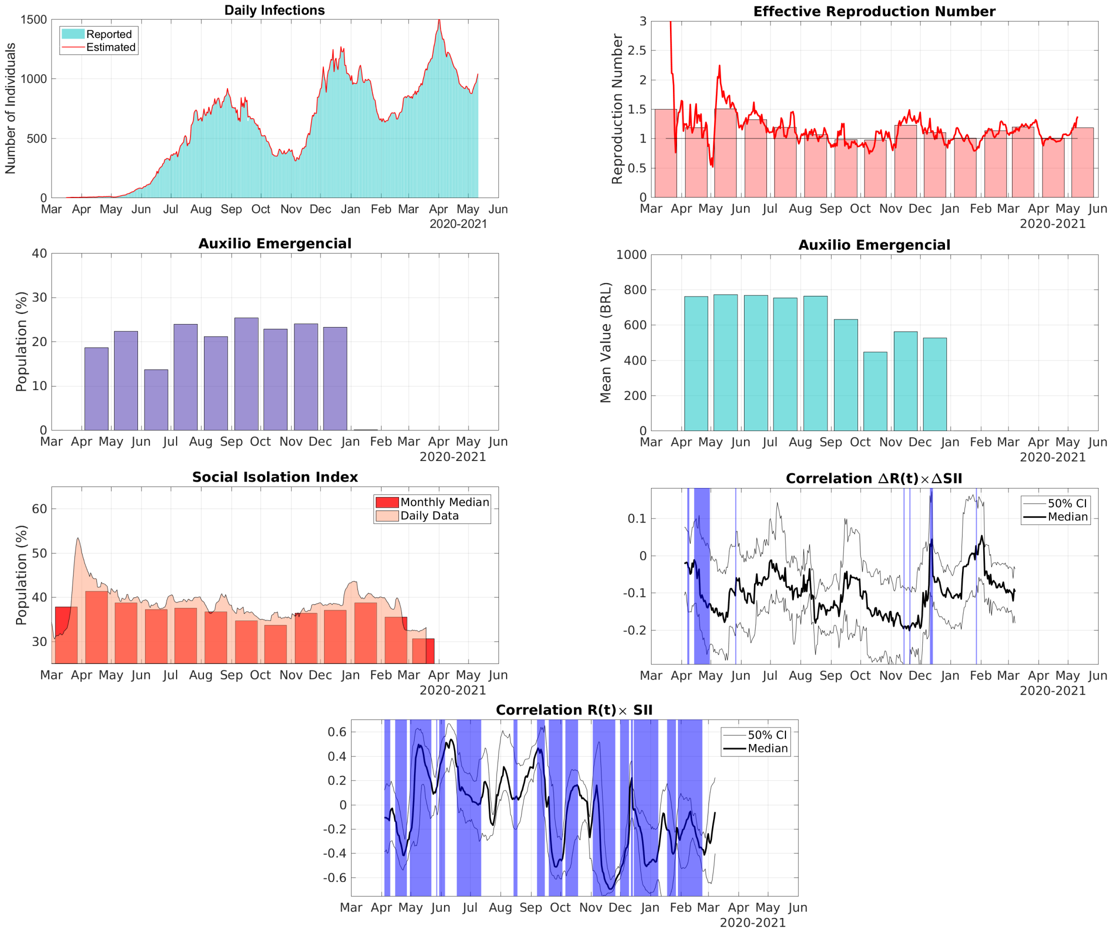

Figure S.20: Data for the State of Mato Grosso do Sul in the Central-West Region of Brazil. Top Row, Right: comparison between the 7-day moving average of daily reports of infections and model predictions (right). Top Row, Left: The solid line represents the time-dependent reproduction number and the bars are the corresponding monthly median values. Middle Row, Right: The bars represent the proportions of the State population receiving the Auxílio Emergencial by month. Middle Row, Left: The bars represent the statewide average amount paid by Auxílio Emergencial each month. Bottom Row, Left: The area graph is the 7-day moving average of the social isolation index and the bars represent the corresponding monthly median values. Bottom Row, Right: Correlation between the daily increments of the social isolation index and the reproduction number. The marked regions in the correlation plot indicate that the estimated values are significantly different from zero, for a 10% p-value.

4 Southeast Region

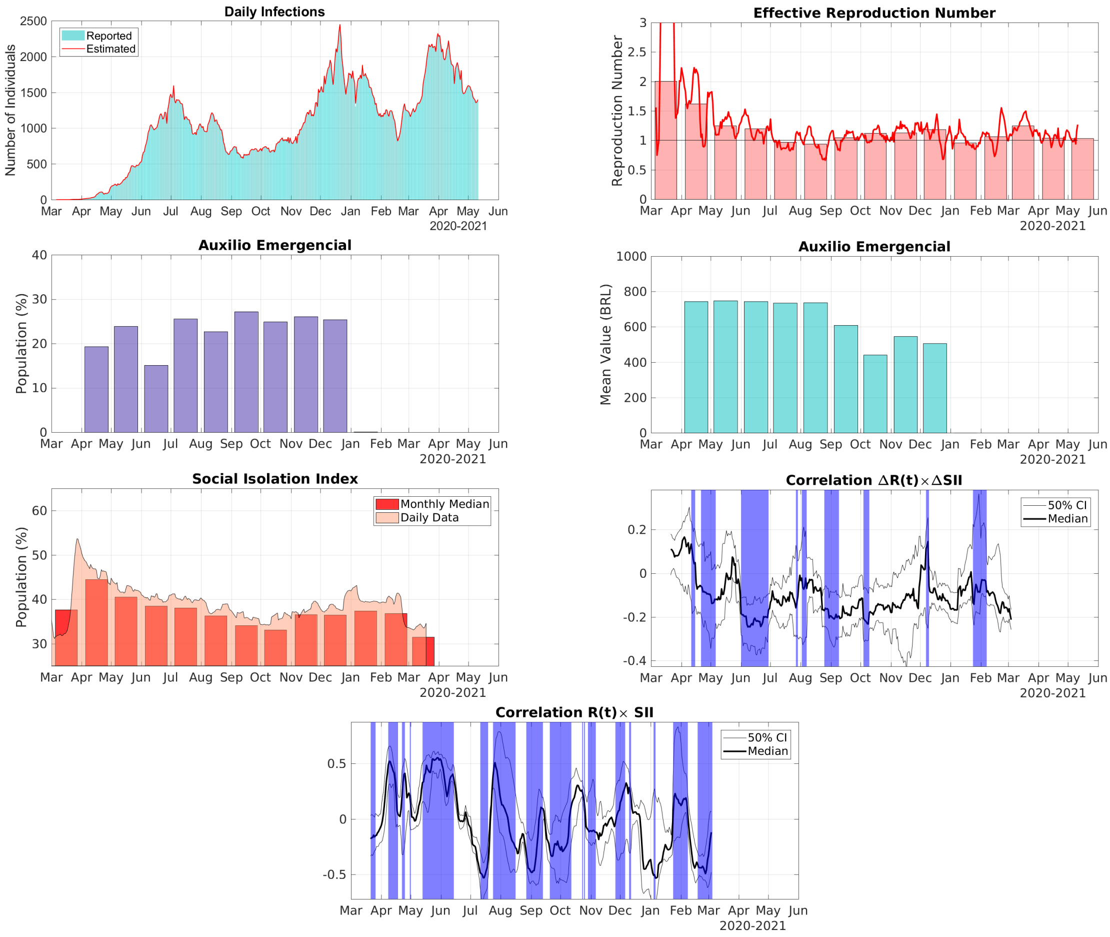

Figure S.21: Data for the State of Espírito Santo in the Southeast Region of Brazil. Top Row, Right: comparison between the 7-day moving average of daily reports of infections and model predictions (right). Top Row, Left: The solid line represents the time-dependent reproduction number and the bars are the corresponding monthly median values. Middle Row, Right: The bars represent the proportions of the State population receiving the Auxílio Emergencial by month. Middle Row, Left: The bars represent the statewide average amount paid by Auxílio Emergencial each month. Bottom Row, Left: The area graph is the 7-day moving average of the social isolation index and the bars represent the corresponding monthly median values. Bottom Row, Right: Correlation between the daily increments of the social isolation index and the reproduction number. The marked regions in the correlation plot indicate that the estimated values are significantly different from zero, for a 10% p-value.

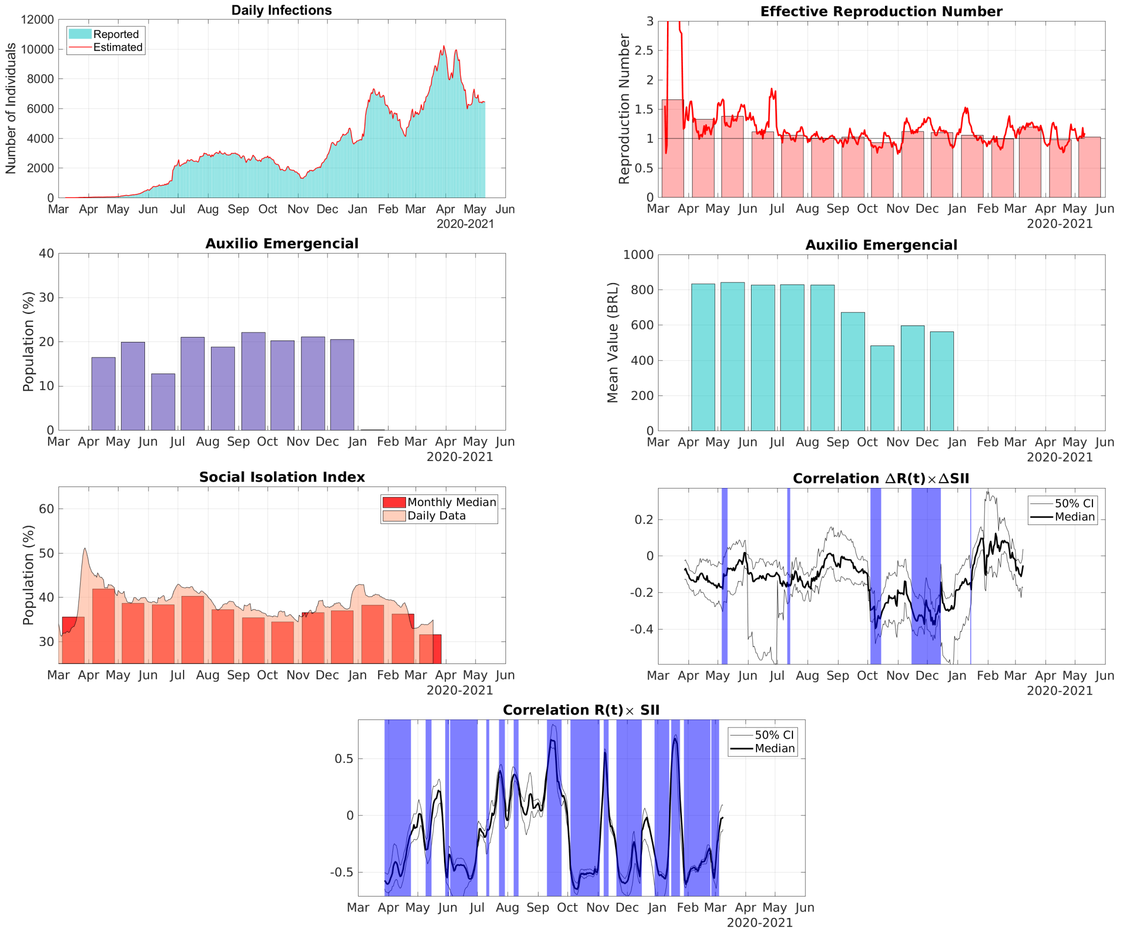

Figure S.22: Data for the State of Minas Gerais in the Southeast Region of Brazil. Top Row, Right: comparison between the 7-day moving average of daily reports of infections and model predictions (right). Top Row, Left: The solid line represents the time-dependent reproduction number and the bars are the corresponding monthly median values. Middle Row, Right: The bars represent the proportions of the State population receiving the Auxílio Emergencial by month. Middle Row, Left: The bars represent the statewide average amount paid by Auxílio Emergencial each month. Bottom Row, Left: The area graph is the 7-day moving average of the social isolation index and the bars represent the corresponding monthly median values. Bottom Row, Right: Correlation between the daily increments of the social isolation index and the reproduction number. The marked regions in the correlation plot indicate that the estimated values are significantly different from zero, for a 10% p-value.

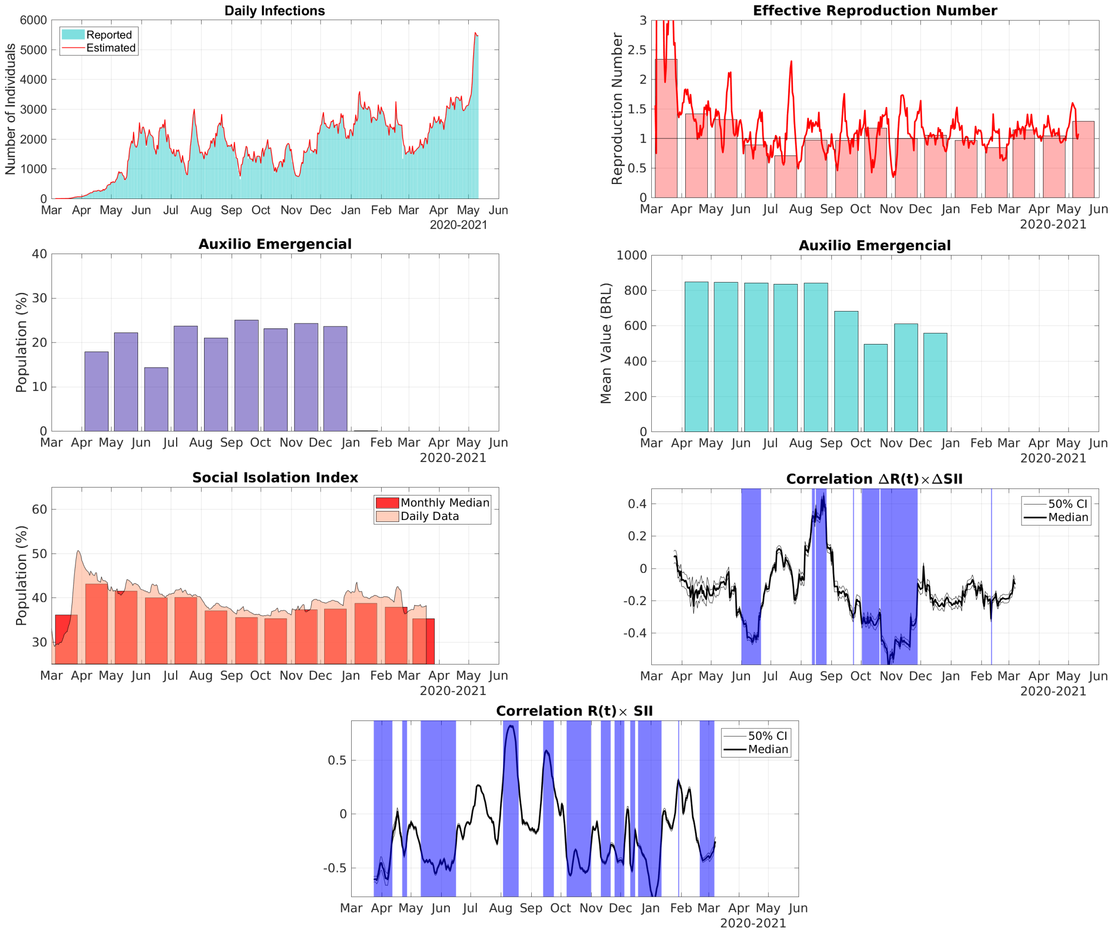

Figure S.23: Data for the State of Rio de Janeiro in the Southeast Region of Brazil. Top Row, Right: comparison between the 7-day moving average of daily reports of infections and model predictions (right). Top Row, Left: The solid line represents the time-dependent reproduction number and the bars are the corresponding monthly median values. Middle Row, Right: The bars represent the proportions of the State population receiving the Auxílio Emergencial by month. Middle Row, Left: The bars represent the statewide average amount paid by Auxílio Emergencial each month. Bottom Row, Left: The area graph is the 7-day moving average of the social isolation index and the bars represent the corresponding monthly median values. Bottom Row, Right: Correlation between the daily increments of the social isolation index and the reproduction number. The marked regions in the correlation plot indicate that the estimated values are significantly different from zero, for a 10% p-value.

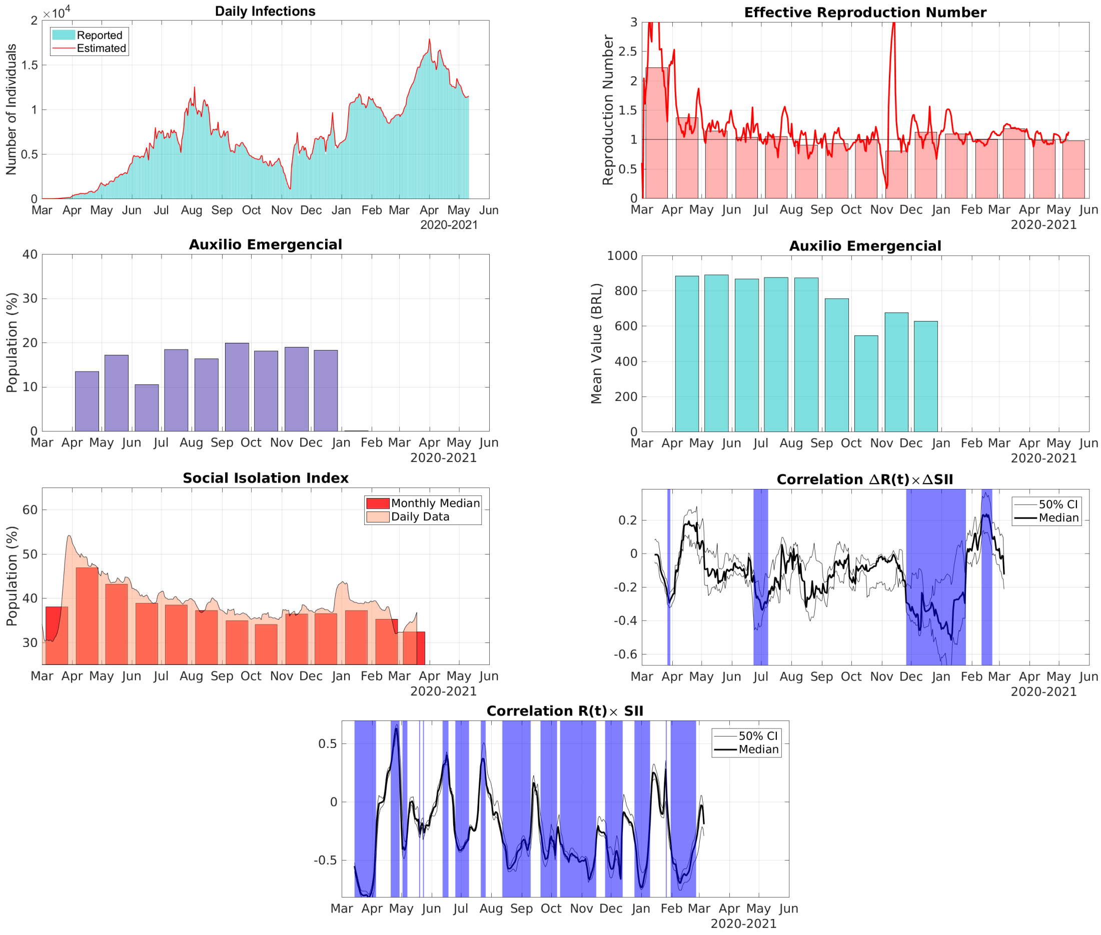

Figure S.24: Data for the State of São Paulo in the Southeast Region of Brazil. Top Row, Right: comparison between the 7-day moving average of daily reports of infections and model predictions (right). Top Row, Left: The solid line represents the time-dependent reproduction number and the bars are the corresponding monthly median values. Middle Row, Right: The bars represent the proportions of the State population receiving the Auxílio Emergencial by month. Middle Row, Left: The bars represent the statewide average amount paid by Auxílio Emergencial each month. Bottom Row, Left: The area graph is the 7-day moving average of the social isolation index and the bars represent the corresponding monthly median values. Bottom Row, Right: Correlation between the daily increments of the social isolation index and the reproduction number. The marked regions in the correlation plot indicate that the estimated values are significantly different from zero, for a 10% p-value.

5 South Region

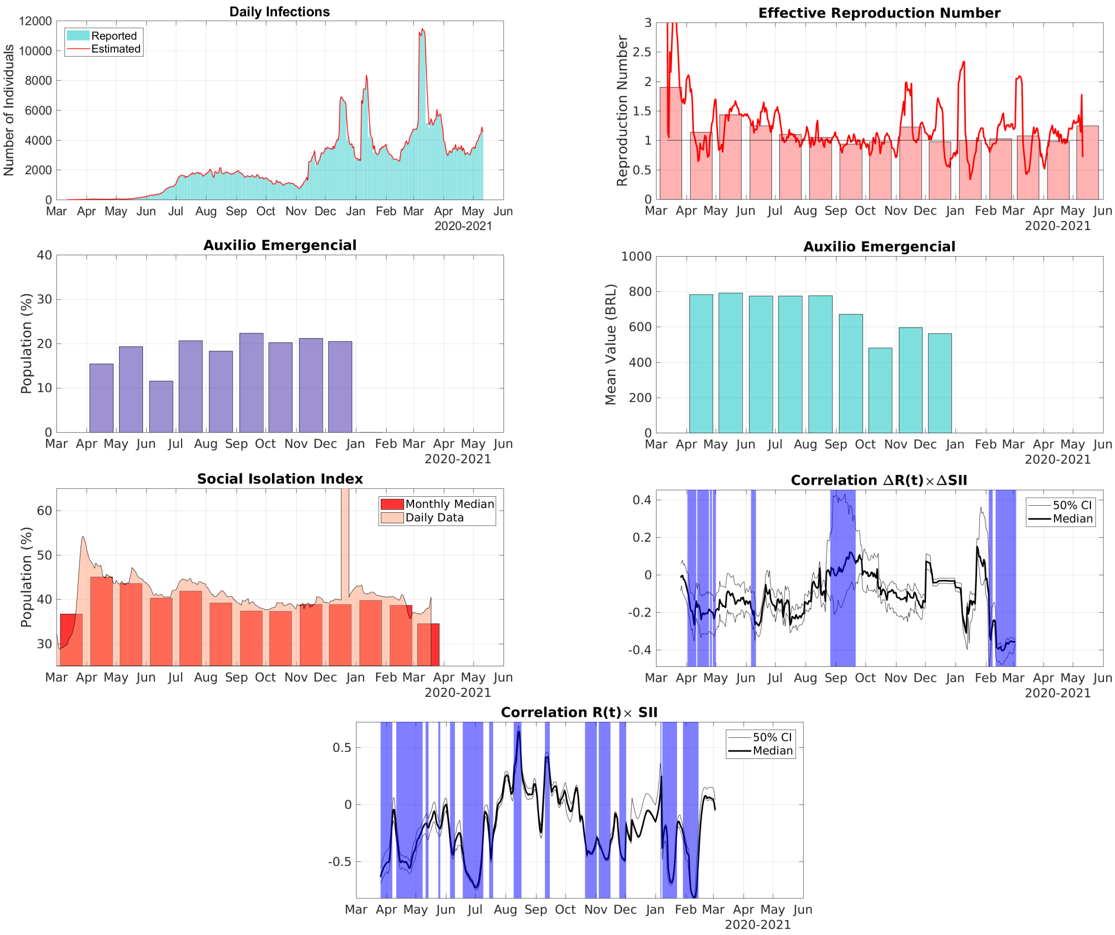

Figure S.25: Data for the State of Paraná in the South Region of Brazil. Top Row, Right: comparison between the 7-day moving average of daily reports of infections and model predictions (right). Top Row, Left: The solid line represents the time-dependent reproduction number and the bars are the corresponding monthly median values. Middle Row, Right: The bars represent the proportions of the State population receiving the Auxílio Emergencial by month. Middle Row, Left: The bars represent the statewide average amount paid by Auxílio Emergencial each month. Bottom Row, Left: The area graph is the 7-day moving average of the social isolation index and the bars represent the corresponding monthly median values. Bottom Row, Right: Correlation between the daily increments of the social isolation index and the reproduction number. The marked regions in the correlation plot indicate that the estimated values are significantly different from zero, for a 10% p-value.

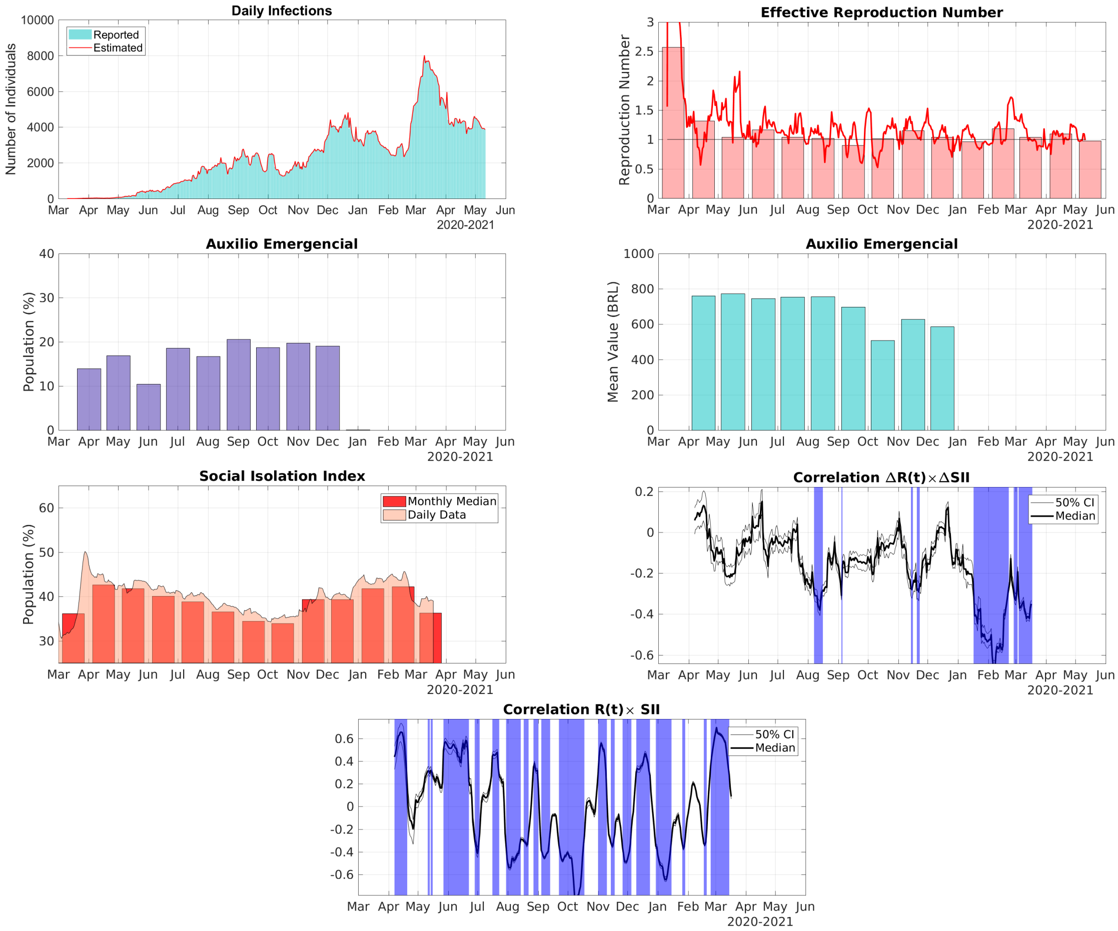

Figure S.26: Data for the State of Rio Grande do Sul in the South Region of Brazil. Top Row, Right: comparison between the 7-day moving average of daily reports of infections and model predictions (right). Top Row, Left: The solid line represents the time-dependent reproduction number and the bars are the corresponding monthly median values. Middle Row, Right: The bars represent the proportions of the State population receiving the Auxílio Emergencial by month. Middle Row, Left: The bars represent the statewide average amount paid by Auxílio Emergencial each month. Bottom Row, Left: The area graph is the 7-day moving average of the social isolation index and the bars represent the corresponding monthly median values. Bottom Row, Right: Correlation between the daily increments of the social isolation index and the reproduction number. The marked regions in the correlation plot indicate that the estimated values are significantly different from zero, for a 10% p-value.

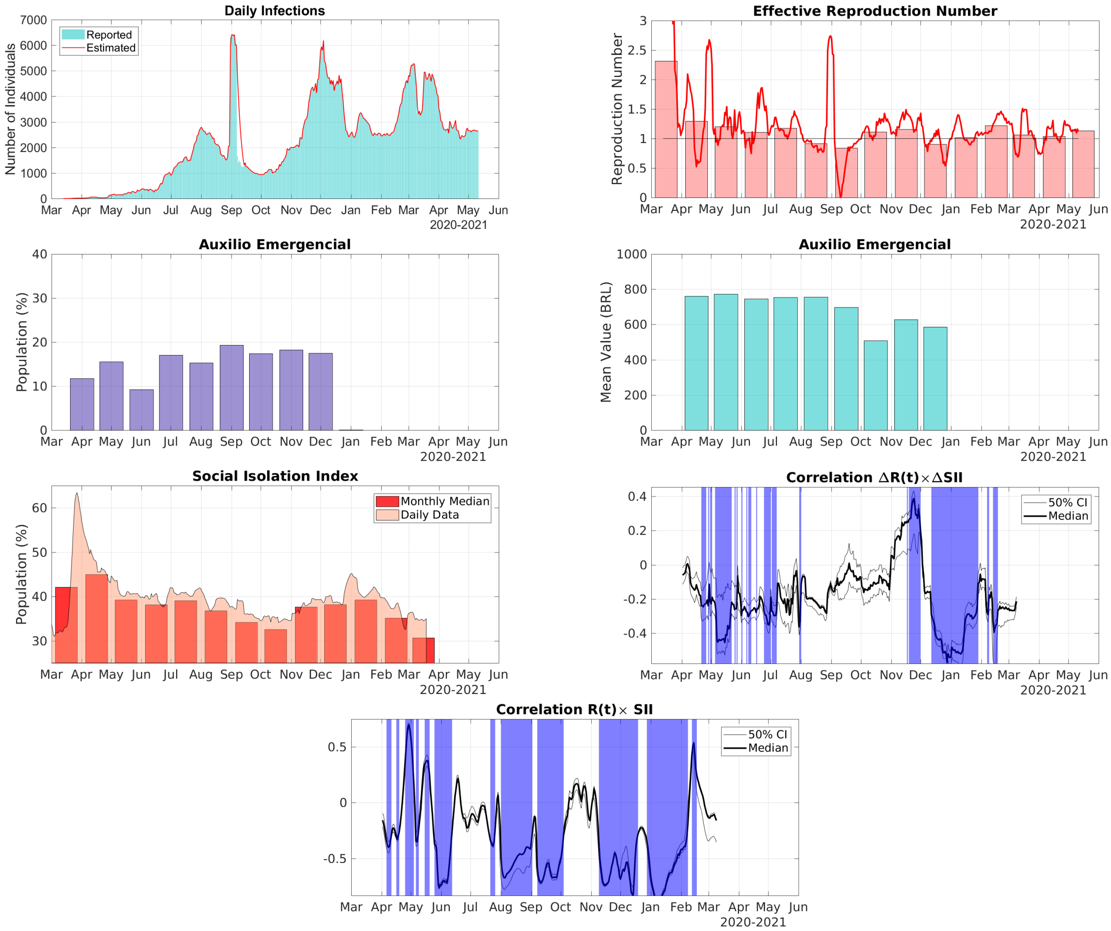

Figure S.27: Data for the State of Santa Catarina in the South Region of Brazil. Top Row, Right: comparison between the 7-day moving average of daily reports of infections and model predictions (right). Top Row, Left: The solid line represents the time-dependent reproduction number and the bars are the corresponding monthly median values. Middle Row, Right: The bars represent the proportions of the State population receiving the Auxílio Emergencial by month. Middle Row, Left: The bars represent the statewide average amount paid by Auxílio Emergencial each month. Bottom Row, Left: The area graph is the 7-day moving average of the social isolation index and the bars represent the corresponding monthly median values. Bottom Row, Right: Correlation between the daily increments of the social isolation index and the reproduction number. The marked regions in the correlation plot indicate that the estimated values are significantly different from zero, for a 10% p-value.

6 Additional Tables

State	Region	Mar-20	Apr-20	May-20	Jun-20	Jul-20	Aug-20	Sep-20	Oct-20	Nov-20	Dec-20	Jan-21	Feb-21	Mar-21	Apr-21	May-21
AC	N	3,06	1,52	1,36	1,02	0,99	0,98	1,03	1,00	1,19	1,06	1,22	1,15	1,10	0,84	0,89
AL	NE	1,10	2,09	1,42	1,06	0,96	0,81	0,91	0,90	1,15	1,16	1,11	0,95	1,17	1,08	1,12
AM	N	2,38	1,68	1,30	0,93	1,11	1,01	1,07	0,96	0,96	1,10	1,19	0,94	1,08	1,02	1,05
AP	N	1,39	1,82	1,44	1,00	0,93	1,13	0,95	1,07	1,26	1,07	1,11	1,08	1,34	0,90	1,20
BA	NE	1,58	1,30	1,28	1,23	1,00	1,00	0,92	1,04	1,18	1,09	1,04	1,12	1,01	1,05	1,09
CE	NE	1,78	1,54	1,26	0,99	1,02	0,89	0,88	0,97	1,07	0,93	1,13	1,02	1,12	0,95	1,05
DF	CW	1,93	1,06	1,41	1,37	1,05	1,00	0,95	1,04	1,06	1,16	1,16	1,18	1,16	1,04	1,12
ES	SE	2,00	1,62	1,25	1,19	0,97	0,94	1,05	1,12	1,13	1,19	0,96	1,07	1,25	1,05	1,03
GO	CW	1,48	1,51	1,24	1,29	1,05	1,05	0,98	0,84	0,97	0,98	1,13	1,10	1,15	1,06	0,89
MA	NE	3,00	1,62	1,52	0,92	1,09	1,01	0,92	0,91	1,04	1,01	1,11	1,13	1,13	1,03	1,04
MG	SE	1,66	1,33	1,38	1,11	1,06	1,01	1,03	0,93	1,12	1,11	1,06	0,99	1,20	0,99	1,03
MS	CW	1,50	1,19	1,51	1,32	1,19	1,07	0,98	0,98	1,23	1,11	1,01	1,14	1,20	1,01	1,19
MT	CW	2,26	1,06	1,54	1,37	1,17	1,05	0,96	0,95	1,07	1,14	1,15	1,11	1,19	1,04	1,04
PA	N	3,05	1,76	1,50	1,02	0,91	1,03	0,90	0,93	1,00	1,05	1,20	1,07	1,09	1,01	1,02
PB	NE	1,56	1,67	1,55	1,11	1,01	0,93	0,94	1,04	1,12	1,15	1,15	1,11	1,08	1,03	1,10
PE	NE	1,16	1,91	1,13	0,97	1,07	0,93	0,89	1,01	1,14	1,10	1,07	0,99	1,02	1,10	1,14
PI	NE	2,02	1,74	1,26	1,25	1,07	0,95	0,98	1,00	1,08	1,09	1,08	1,09	1,21	1,04	1,08
PR	S	1,90	1,14	1,44	1,25	1,10	1,05	0,94	0,98	1,23	0,98	1,00	1,03	1,08	0,98	1,25
RJ	SE	2,34	1,42	1,32	0,89	0,71	0,98	0,97	1,18	1,00	1,06	0,97	0,85	1,15	1,05	1,30
RN	NE	1,86	1,27	1,22	1,31	0,72	0,93	0,95	1,00	1,27	1,14	1,04	0,96	1,12	1,24	1,05
RO	N	1,40	1,96	1,41	1,31	0,78	0,99	0,91	0,90	1,26	1,15	1,21	1,07	1,16	1,02	1,14
RR	N	2,25	1,55	1,19	1,37	0,91	1,03	1,09	1,14	0,91	0,88	1,33	1,23	1,10	1,26	1,28
RS	S	2,57	1,31	1,04	1,17	1,04	1,02	0,90	1,01	1,15	1,04	0,96	1,18	1,04	1,10	0,98
SC	S	2,32	1,29	1,20	1,11	1,18	0,92	0,84	1,11	1,16	0,91	1,02	1,23	1,07	1,05	1,15
SE	NE	1,03	1,68	1,44	1,22	1,12	0,83	0,96	1,15	1,41	1,27	1,09	1,01	1,30	1,03	1,06
SP	SE	2,23	1,38	1,15	1,04	1,05	0,91	0,93	0,99	0,81	1,13	1,10	1,01	1,18	0,99	0,98
TO	N	1,24	1,36	1,54	1,03	1,11	1,11	0,92	0,86	1,16	1,07	1,08	1,12	1,18	1,05	1,12

Table S.1: Monthly median values of the reproduction number in the Brazilian states.

State	Region	Apr-20	May-20	Jun-20	Jul-20	Aug-20	Sep-20	Oct-20	Nov-20	Dec-20
AC	N	R$ 752,28	R$ 755,60	R$ 757,13	R$ 747,78	R$ 750,59	R$ 501,52	R$ 365,14	R$ 444,98	R$ 416,38
AL	NE	R$ 985,97	R$ 982,85	R$ 993,75	R$ 970,99	R$ 974,97	R$ 627,79	R$ 461,30	R$ 556,13	R$ 528,66
AM	N	R$ 780,90	R$ 780,21	R$ 786,16	R$ 772,84	R$ 778,24	R$ 545,09	R$ 400,33	R$ 483,18	R$ 451,91
AP	N	R$ 778,26	R$ 775,91	R$ 795,34	R$ 766,05	R$ 770,25	R$ 558,66	R$ 397,73	R$ 491,10	R$ 463,51
BA	NE	R$ 937,55	R$ 941,72	R$ 940,22	R$ 931,73	R$ 932,72	R$ 612,42	R$ 445,48	R$ 542,90	R$ 519,31
CE	NE	R$ 846,61	R$ 848,23	R$ 851,73	R$ 839,80	R$ 841,70	R$ 548,83	R$ 399,18	R$ 484,63	R$ 464,09
DF	CW	R$ 769,64	R$ 769,37	R$ 762,81	R$ 754,89	R$ 761,52	R$ 662,92	R$ 482,11	R$ 593,56	R$ 557,28
ES	SE	R$ 744,43	R$ 748,46	R$ 744,19	R$ 735,21	R$ 736,41	R$ 608,66	R$ 441,69	R$ 546,38	R$ 506,08
GO	CW	R$ 801,76	R$ 807,14	R$ 797,68	R$ 791,64	R$ 792,03	R$ 671,92	R$ 483,87	R$ 594,82	R$ 562,63
MA	NE	R$ 866,46	R$ 871,20	R$ 877,13	R$ 858,27	R$ 861,45	R$ 531,70	R$ 390,30	R$ 467,19	R$ 458,47
MG	SE	R$ 834,10	R$ 842,36	R$ 827,68	R$ 829,24	R$ 828,48	R$ 671,52	R$ 482,11	R$ 597,12	R$ 562,48
MS	CW	R$ 762,56	R$ 773,00	R$ 768,47	R$ 755,02	R$ 765,51	R$ 632,85	R$ 446,42	R$ 562,13	R$ 526,91
MT	CW	R$ 778,40	R$ 788,24	R$ 782,33	R$ 769,53	R$ 771,59	R$ 649,78	R$ 461,15	R$ 575,00	R$ 544,76
PA	N	R$ 809,95	R$ 813,07	R$ 819,26	R$ 805,00	R$ 807,85	R$ 547,54	R$ 396,53	R$ 482,84	R$ 463,57
PB	NE	R$ 859,16	R$ 859,63	R$ 865,49	R$ 850,28	R$ 853,08	R$ 533,89	R$ 394,13	R$ 475,56	R$ 450,14
PE	NE	R$ 990,51	R$ 988,95	R$ 999,31	R$ 976,53	R$ 983,44	R$ 664,51	R$ 474,76	R$ 577,65	R$ 548,07
PI	NE	R$ 851,90	R$ 855,71	R$ 856,36	R$ 846,75	R$ 848,35	R$ 522,08	R$ 382,96	R$ 461,44	R$ 446,42
PR	S	R$ 783,62	R$ 792,75	R$ 774,35	R$ 775,62	R$ 777,08	R$ 672,41	R$ 480,79	R$ 597,13	R$ 562,76
RJ	SE	R$ 848,42	R$ 846,16	R$ 843,03	R$ 835,77	R$ 841,75	R$ 682,27	R$ 496,24	R$ 610,48	R$ 558,86
RN	NE	R$ 832,20	R$ 833,94	R$ 837,32	R$ 824,10	R$ 829,27	R$ 564,98	R$ 406,54	R$ 498,13	R$ 472,49
RO	N	R$ 743,91	R$ 755,35	R$ 746,47	R$ 738,25	R$ 740,95	R$ 620,22	R$ 438,58	R$ 547,53	R$ 515,04
RR	N	R$ 748,40	R$ 753,87	R$ 750,43	R$ 739,63	R$ 746,65	R$ 564,87	R$ 412,72	R$ 510,46	R$ 469,91
RS	S	R$ 771,56	R$ 777,97	R$ 769,12	R$ 763,45	R$ 774,51	R$ 681,96	R$ 475,20	R$ 593,87	R$ 557,20
SC	S	R$ 761,53	R$ 773,25	R$ 745,64	R$ 754,56	R$ 756,38	R$ 698,06	R$ 507,44	R$ 627,98	R$ 586,93
SE	NE	R$ 914,49	R$ 913,83	R$ 924,91	R$ 904,31	R$ 908,71	R$ 594,17	R$ 433,47	R$ 527,89	R$ 504,25
SP	SE	R$ 885,30	R$ 891,15	R$ 868,12	R$ 876,77	R$ 873,79	R$ 755,58	R$ 546,93	R$ 675,85	R$ 627,22
TO	N	R$ 753,58	R$ 761,15	R$ 767,12	R$ 748,91	R$ 751,80	R$ 562,60	R$ 392,69	R$ 484,97	R$ 475,22

Table S.2: Monthly average amount paid by Auxílio Emergencial during 2020 for each Brazilian state.

State	Region	Feb-20	Mar-20	Apr-20	May-20	Jun-20	Jul-20	Aug-20	Sep-20	Oct-20	Nov-20	Dec-20	Jan-21	Feb-21	Mar-21
AC	N	31,2%	43,5%	47,0%	48,1%	44,0%	43,0%	41,1%	38,6%	37,8%	42,8%	42,7%	45,8%	45,3%	38,7%
AL	NE	25,8%	35,4%	43,7%	44,0%	40,6%	40,2%	37,6%	35,9%	35,3%	36,7%	36,5%	38,0%	37,2%	34,0%
AM	N	27,8%	42,6%	47,7%	51,8%	43,7%	41,6%	38,7%	37,3%	36,2%	39,9%	39,8%	42,9%	42,0%	36,3%
AP	N	30,5%	43,2%	52,3%	48,1%	40,8%	40,6%	39,3%	37,2%	36,7%	41,7%	42,4%	53,0%	48,2%	40,1%
BA	NE	26,5%	37,6%	43,9%	42,9%	39,8%	41,0%	38,5%	36,6%	35,8%	36,9%	36,9%	38,2%	38,0%	37,1%
CE	NE	28,3%	41,9%	48,3%	48,7%	41,2%	41,3%	39,4%	37,8%	37,6%	40,4%	40,2%	41,6%	40,8%	41,9%
DF	CW	26,5%	39,8%	47,1%	42,4%	39,8%	40,2%	38,7%	36,3%	35,1%	38,5%	38,2%	39,1%	37,9%	36,3%
ES	SE	27,4%	37,6%	44,5%	40,6%	38,5%	38,1%	36,3%	34,1%	33,1%	36,6%	36,5%	37,4%	36,8%	31,5%
GO	CW	26,3%	37,8%	42,0%	37,4%	35,7%	37,1%	35,1%	33,6%	32,1%	34,8%	35,2%	35,6%	34,3%	33,0%
MA	NE	27,8%	36,9%	44,3%	47,6%	38,2%	38,7%	37,2%	36,1%	35,6%	38,5%	38,4%	40,3%	39,5%	36,6%
MG	SE	28,2%	35,6%	41,9%	38,6%	38,3%	40,2%	37,2%	35,4%	34,4%	36,6%	36,9%	38,3%	36,3%	31,6%
MS	CW	28,5%	37,8%	41,4%	38,7%	37,3%	37,6%	36,7%	34,7%	33,7%	36,4%	37,1%	38,8%	35,5%	30,6%
MT	CW	27,0%	36,8%	43,6%	39,5%	36,7%	37,6%	36,1%	34,2%	33,2%	35,4%	35,9%	36,8%	35,6%	32,7%
PA	N	27,4%	37,4%	44,3%	39,4%	37,9%	38,6%	37,1%	34,1%	32,8%	36,8%	36,8%	38,3%	35,5%	34,3%
PB	NE	26,5%	36,9%	44,5%	44,0%	40,5%	40,0%	37,5%	36,0%	35,8%	38,5%	38,4%	39,5%	38,9%	35,6%
PE	NE	28,1%	37,9%	46,2%	48,6%	38,8%	38,2%	37,4%	35,6%	34,9%	38,9%	39,0%	41,5%	41,4%	36,8%
PI	NE	26,5%	39,6%	48,2%	47,3%	40,9%	40,0%	38,1%	36,8%	36,0%	37,7%	37,7%	38,6%	38,0%	33,5%
PR	S	26,4%	36,7%	45,1%	43,6%	40,3%	41,9%	39,2%	37,4%	37,3%	38,9%	38,8%	39,8%	38,7%	34,5%
RJ	SE	26,2%	36,1%	43,1%	41,5%	40,0%	40,1%	37,1%	35,6%	35,3%	37,3%	37,4%	38,8%	37,9%	35,3%
RN	NE	28,1%	39,8%	47,3%	41,6%	38,8%	40,3%	37,7%	35,4%	34,3%	35,9%	36,5%	36,6%	35,2%	34,1%
RO	N	29,2%	41,1%	49,5%	46,6%	41,3%	40,3%	38,6%	37,1%	35,8%	39,3%	39,9%	40,6%	39,2%	33,2%
RR	N	29,4%	38,7%	44,6%	43,0%	42,0%	41,3%	38,9%	36,9%	36,4%	40,3%	40,5%	46,2%	42,3%	37,6%
RS	S	27,3%	36,2%	42,7%	41,8%	40,1%	38,8%	36,6%	34,4%	33,9%	39,4%	39,3%	41,8%	42,2%	36,3%
SC	S	28,5%	42,1%	45,0%	39,3%	38,1%	39,1%	36,8%	34,2%	32,6%	37,6%	38,2%	39,3%	35,2%	30,7%
SE	NE	26,0%	36,7%	42,3%	41,6%	38,3%	40,1%	37,5%	35,2%	34,2%	37,3%	36,7%	38,1%	37,2%	35,0%
SP	SE	26,5%	38,1%	46,9%	43,2%	38,9%	38,5%	37,3%	35,0%	34,1%	36,5%	36,6%	37,3%	35,3%	32,4%
TO	N	24,6%	38,3%	38,4%	37,5%	34,4%	35,1%	34,7%	33,2%	31,3%	33,4%	33,8%	34,9%	33,5%	32,5%

Table S.3: Monthly median values of the social isolation index (SII).

State	Region	Feb-20	Mar-20	Apr-20	May-20	Jun-20	Jul-20	Aug-20	Sep-20	Oct-20	Nov-20	Dec-20	Jan-21	Feb-21	Mar-21	Apr-21
AC	N	0,00	4,70	40,5	650	786	712	561	400	288	611	599	765	1014	1355	911
AL	NE	0,00	0,54	30,6	276	766	709	569	241	117	125	293	386	417	649	602
AM	N	0,00	4,16	121	859	700	716	460	452	531	393	542	1578	1154	788	515
AP	N	0,00	1,16	124	989	2192	926	780	587	455	807	1052	1026	768	1611	953
BA	NE	0,00	1,43	17,7	104	368	622	607	360	286	334	605	634	643	801	651
CE	NE	0,00	4,25	78,5	445	655	710	447	285	359	288	375	417	566	1229	1407
DF	CW	0,00	10,9	33,5	276	1291	1868	1825	989	687	520	738	832	641	1560	1127
ES	SE	0,00	2,07	58,6	276	817	896	688	498	596	841	1436	1128	792	1375	1339
GO	CW	0,00	0,91	10,1	41,1	291	612	908	1081	647	337	414	586	636	1244	944
MA	NE	0,00	0,44	44,4	442	644	565	435	309	172	103	109	92	166	325	344
MG	SE	0,00	1,29	7,29	40,6	162	386	420	369	300	269	594	900	677	1152	1105
MS	CW	0,00	1,71	7,37	43,9	231	604	854	739	450	595	1235	970	730	1213	1160
MT	CW	0,00	0,71	7,71	60,5	375	1027	1120	909	553	432	604	1032	953	1677	1457
PA	N	0,00	0,37	32,7	404	751	592	516	355	259	203	265	412	407	608	622
PB	NE	0,00	0,42	19,7	306	837	887	569	383	294	300	526	621	731	942	829
PE	NE	0,00	0,90	70,6	287	254	376	318	225	163	203	413	407	397	517	596
PI	NE	0,00	0,55	15,1	135	472	946	790	569	524	418	477	498	447	954	1079
PR	S	0,00	1,55	10,7	28,5	158	462	484	408	300	578	1185	1153	848	1729	882
RJ	SE	0,00	4,08	50,4	253	341	305	335	237	260	256	462	497	357	373	546
RN	NE	0,00	2,32	31,0	176	648	570	321	217	332	395	650	637	745	831	756
RO	N	0,00	0,45	27,5	247	908	988	900	599	320	494	846	1611	1342	2143	1418
RR	N	0,00	2,53	79,7	468	1769	2753	1822	1098	1083	957	850	860	1257	1184	1076
RS	S	0,00	2,40	10,4	68,9	154	348	518	567	499	652	1117	854	820	1790	1134
SC	S	0,00	3,02	25,7	95,9	239	796	1292	520	599	1453	1768	1161	1293	1880	1117
SE	NE	0,00	0,82	18,5	283	794	1436	596	212	297	251	964	1074	603	1000	1172
SP	SE	0,00	5,05	56,9	175	371	564	566	392	282	271	477	681	571	925	937
TO	N	0,00	0,69	7,92	254	414	884	1627	1088	476	381	549	746	736	1701	1178

Table S.4: Number of COVID-19 infections per 100K individuals by month for each Brazilian state.
